# Climate Change Impacts on the Upper Indus Hydrology: Sources, Shifts and Extremes

**DOI:** 10.1371/journal.pone.0165630

**Published:** 2016-11-09

**Authors:** A. F. Lutz, W. W. Immerzeel, P. D. A. Kraaijenbrink, A. B. Shrestha, M. F. P. Bierkens

**Affiliations:** 1 FutureWater, Costerweg 1V, 6702 AA, Wageningen, The Netherlands; 2 Utrecht University, Department of Physical Geography, PO Box 80115, 3508 TC, Utrecht, The Netherlands; 3 International Centre for Integrated Mountain Development, GPO Box 3226, Khumaltar, Kathmandu, Nepal; Universidade de Vigo, SPAIN

## Abstract

The Indus basin heavily depends on its upstream mountainous part for the downstream supply of water while downstream demands are high. Since downstream demands will likely continue to increase, accurate hydrological projections for the future supply are important. We use an ensemble of statistically downscaled CMIP5 General Circulation Model outputs for RCP4.5 and RCP8.5 to force a cryospheric-hydrological model and generate transient hydrological projections for the entire 21^st^ century for the upper Indus basin. Three methodological advances are introduced: (i) A new precipitation dataset that corrects for the underestimation of high-altitude precipitation is used. (ii) The model is calibrated using data on river runoff, snow cover and geodetic glacier mass balance. (iii) An advanced statistical downscaling technique is used that accounts for changes in precipitation extremes. The analysis of the results focuses on changes in sources of runoff, seasonality and hydrological extremes. We conclude that the future of the upper Indus basin’s water availability is highly uncertain in the long run, mainly due to the large spread in the future precipitation projections. Despite large uncertainties in the future climate and long-term water availability, basin-wide patterns and trends of seasonal shifts in water availability are consistent across climate change scenarios. Most prominent is the attenuation of the annual hydrograph and shift from summer peak flow towards the other seasons for most ensemble members. In addition there are distinct spatial patterns in the response that relate to monsoon influence and the importance of meltwater. Analysis of future hydrological extremes reveals that increases in intensity and frequency of extreme discharges are very likely for most of the upper Indus basin and most ensemble members.

## Introduction

**Fig 1:**
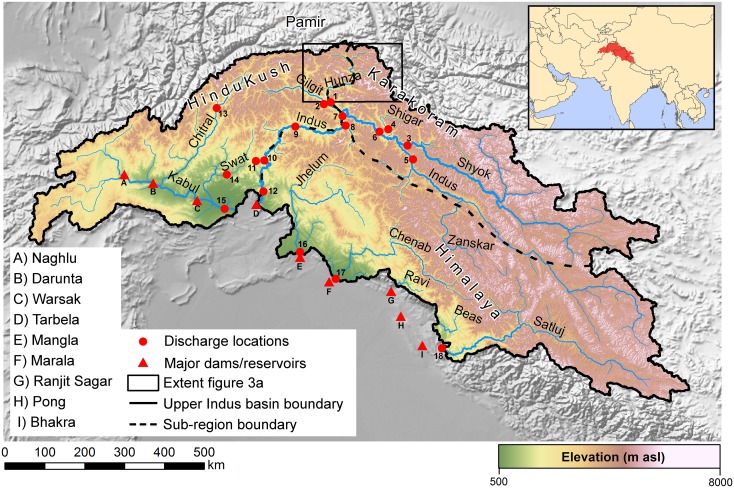
The upper Indus basin. The map shows the main rivers, mountain ranges, digital elevation model [18] and locations of the main dams. Numbered red dots indicate stream flow locations referred to in other figures. Source of the background hillshade is www.naturalearthdata.com. Source of political borders displayed in the inset is the GADM database of Global Administrative Areas (www.gadm.org).

The water resources supplied by the upper Indus basin (UIB) are essential to millions of people and future changes in both demand and supply may have large impacts [1]. The UIB provides water for the world's largest continuous irrigation scheme through several large reservoirs (e.g. the Tarbela and Mangla dams, Fig 1), which depend for more than 50% of their annual inflow on snow and glacier melt water [2–5]. In combination with variable precipitation patterns, the intra-annual variation in streamflow is high [6] and so is the supply to the downstream areas. Water demands are high, primarily because of water consumption by irrigated agriculture [7], and hydropower generation [8]. At the same time the downstream part of the basin is characterized by very dry conditions [9], making it largely dependent on water supply from the upstream areas. The downstream demands exceed the supply and on an annual basis groundwater resources are depleted by an estimated 31 km^3^ [10], which makes the Indus basin aquifer the most overstressed aquifer in the world [11,12]. The uncertainty in the future mountain water resources combined with the Indus basin’s large dependency on these upstream water resources, makes the Indus basin a climate change hotspot [1,13]. This vulnerability is enhanced by an expected large regional population growth in the coming decades [14], associated with increases in water and energy demand in the basin. Extreme weather events are likely to become more frequent in the region in the future [15,16], which poses serious threats for a region which is already facing severe flooding events [17] and other natural hazards.

The UIB has a complex climate. Several studies investigated historical trends in precipitation and temperature in the UIB. Trend analysis on precipitation for 17 stations throughout the UIB showed statistically significant increasing trends in precipitation for several stations in annual, summer and winter precipitation between 1961 and 1990 [19]. Air temperature trends between 1961 and 2000 were also assessed and it was found that (i) the diurnal temperature range is increasing consistently in all seasons, (ii) winter mean and maximum temperatures show significant increases and (iii) mean and minimum summer temperatures show a consistent declining trend [20]. These findings were confirmed also for a more recent period (1980–2009) for roughly the same stations [21]. Trend analysis on the ERA40 reanalysis dataset for the Baltoro region in the Karakoram (Fig 1) showed negative summer temperature trends from 1958 until 1990 and a positive trend from 1991 to 2001 [22]. The authors also found an increasing trend in annual precipitation from 1970 to 1990 and a decreasing trend during the 1990s. Trend analysis on several gridded precipitation products did not confirm these findings [23]. Studies on the winter westerly disturbances, being the major source of winter precipitation, indicate strong intra-seasonal variability and a trend of enhanced frequency and strength of these disturbances in the Karakoram and western Himalaya between 1979 and 2010, leading to increased heavy winter precipitation [24,25].

There is great debate on the response of glaciers in the UIB to climate change during the last decade. The glaciers in the Himalayan range are seemingly losing mass at rates similar to other mountainous regions in the world, however the glaciers in the Karakoram and Pamir mountain ranges have neutral mass balances on average and are characterized by a large number of surging glaciers [26–31]. This so called Karakoram anomaly has not been explained, but a possible reason could be a combination of a decrease in summer temperatures and an increase in precipitation. However this is still speculative and requires further study and understanding of atmospheric processes leading to high-altitude precipitation. This hypothesis is supported by an increasing trend in snow cover that was found in the Hunza basin based on MODIS snow cover analysis [32,33] and that the water balance of the UIB can be closed without large negative glacier mass balances [6]. On the other hand, decreasing trends in snow cover for the most westerly-influenced subbasins, including Hunza, and increasing trends for the more monsoon-influenced subbasins were found [34]. A trend analysis of snow cover in the monsoon-dominated Sutlej basin indicated a trend of snow cover reduction between 2000 and 2009 [35]. Other research concludes that the Karakoram is protected from reductions in annual snowfall under climatic warming because the seasonal cycle is dominated by non-monsoonal winter precipitation [36].

Rising temperatures in basins strongly dependent on glacier melt are likely to result in an increase in stream flow in the near future and a decline in the far future. This is caused by the fact that the total amount of glacier melt is a trade-off between increasing melt rates on one hand and reduced glacier volumes on the other hand. The moment when the trend in glacier melt changes from positive to negative is highly variable [37,38]. Analysis of a 1961 to 2009 record of reservoir inflow at Tarbela, which is the largest reservoir on the main stem of the Indus river (Fig 1), indicates a declining trend, although statistically insignificant [6]. Further upstream trend analysis on streamflow records at different locations identified stable or declining trends in runoff too [32,39,40]. These studies indicate that large parts of the UIB are (not yet) experiencing accelerated melt, which could indeed be partly attributed to the Karakoram anomaly. However, contrary to these findings, a recent study in the Shigar river basin reports rising river flows [41]. However, the authors do not relate this to the existence of the Karakoram anomaly. Instead, they argue that an increase in runoff is possible under neutral glacier mass balance conditions as a result of increasing temperature and precipitation, i.e. the mass turnover of the glacier is increasing, yet the mass balance remains neutral.

Climate simulations are used to generate projections of future climate change in the UIB. Analysis of precipitation change signals in a large number of General Circulation Model (GCM) runs indicates that an increase in summer precipitation and on average no significant change in winter precipitation are likely [42]. However, the spread in the precipitation changes from the GCM ensemble is large, because the complex UIB climate is difficult to simulate [43]. Analysis with regional climate models (RCM) reveals consistent warming until the end of the century with greater warming in the upper Indus than in the lower Indus. Precipitation projections show a non-uniform change with increases projected for the upper parts and decreases for the lower parts [44,45]. However care needs to be taken in using RCMs directly in impact studies. A recent study that analyzed the uncertainty of the CORDEX South Asia regional climate models showed that the RCMs exhibit large uncertainties in both temperature and precipitation, that they exhibit a large cold bias and that they are unable to reproduce observed warming trends [46]. Empirical-statistical downscaling, which may be better suited under such complex conditions, is another approach to generate forcing for climate change impact models, where climate model output is statistically corrected using transfer functions with local observations during a historical period. Empirical-statistical downscaling of GCMs in the UIB based on an ensemble of selected GCMs showed a modest increase in precipitation and a consistent warming, which is stronger in the upper parts of the basin [3,37]. The application of a stochastic weather generator to downscale RCM data in the northern UIB lead to a projection of year-round increasing precipitation, with increased intensity during the wettest months and year-round uniformly increasing temperatures [47].

Hydrological impact studies have been conducted for the UIB at various spatial scales and key assumptions in those studies relate to (i) the reference climate dataset being used, (ii) the future climate forcing and downscaling method, (iii) the type and complexity of the hydrological model, (iv) the treatment of glacier evolution in the future and (v) the calibration and validation strategy. Hydrological projections based on different approaches indicate likely increases in flow at least during the first half of the 21^st^ century for particular subbasins [37,38] and at the basin scale [3,44,45,48]. Projections of changes in hydrological extremes in the UIB are very limited [49], but are at the same time very much desired [3,38,50].

In this study we systematically assess the present day hydrology of the UIB and the impacts of climate change using a new fully distributed cryospheric-hydrological model at a high spatial resolution (1 km^2^) that includes all relevant components of the high altitude water balance [51]. We introduce several novel components which may advance our understanding of the complex impact of climate change on the UIB hydrology:

- A new historical precipitation dataset [52] that corrects for the underestimation of high altitude precipitation is used.
- The model is calibrated on river runoff at several locations, as well as MODIS based snow cover estimates and geodetic glacier mass balance data.
- An advanced statistical downscaling technique for climate change scenarios until 2100 is used that accounts for changes in precipitation extremes.
- The analysis is focusing on changes in sources of runoff, changes in seasonality and changes in hydrological extremes.

## Study area

The upper Indus basin is located in the mountain ranges of the Hindu Kush, Karakoram, Himalaya and on the Tibetan Plateau. Seven different tributaries of the Indus drain from the UIB, covering a surface area of ∼425.000 km^2^ (Fig 1). The UIB covers an altitudinal range of ∼8500 m a.s.l., with a mean elevation of ∼3750 m a.s.l. Covering parts of Afghanistan, Pakistan, India and China makes the UIB a transboundary river basin in a geopolitically complex region. It is among the most glaciated areas on Earth, with ∼22.000 km^2^ of glacier surface area [53].

The climate of the UIB is complex and is the result of an intricate interaction between monsoon circulation, westerlies and the topography [46,54–57]. The interaction between topography and precipitation manifests itself at various scales ranging from a synoptic scale of several hundreds of kilometers to an orographic meso-scale of less than 30 kilometers [58]. Along the Himalayan arc the monsoon influence is largest but this influence decreases in north-western direction where mid-latitude westerlies become increasingly important, e.g. at the junction of the Karakoram, Pamir and Hindu-Kush mountain ranges (Fig 1). Precipitation from the westerlies is highest in winter when low-pressure systems reach the western margin of the greater Himalaya. This supply of moisture reaches higher elevations than the summer monsoon, which might be related to the higher tropospheric extent of the westerly airflow [55].

## Methods

### Cryospheric-hydrological model

We use the high resolution, raster-based, fully distributed Spatial Processes in Hydrology (SPHY) cryospheric-hydrological model (open source, version 2.0) [51], which was applied in a river basin-scale study on climate change impacts for water availability in five major Asian river basins before [3]. The model runs at 1 km^2^ spatial resolution with a daily time step. The actual runoff which is calculated for each grid cell consists of four possible contributing factors: rainfall-runoff, snow melt, glacier melt, and baseflow. For each grid cell the total runoff generated per time step (Q_TOT_) is calculated:

$$Q_{TOT}=Q_{GM}+Q_{SM}+Q_{RR}+Q_{BF}$$ (1)

where Q_GM_ is runoff from glacier melt, Q_SM_ is runoff from snow melt, Q_RR_ is rainfall-runoff and Q_BF_ is baseflow. To determine the contribution of each of the four components to the total runoff within a grid cell, a subgrid parameterization is used in which for each cell the fractional ice cover (G_F_), ranging from 0 (no ice cover) to 1 (complete ice cover), is determined. Glacier melt is simulated using a degree-day modelling approach [59]. A differentiation in debris-covered and debris-free glaciers is made based on thresholds for elevation and terrain slope [60], and different degree day factors are used for both glacier types (Table 1). For the remaining fraction of the grid cell, the model maintains a dynamic snow and soil water storage. The glacier fraction per grid cell is adapted dynamically in time. A variable groundwater storage is maintained for the entire grid cell. A part of the glacier melt generated in the glacierized cell fraction is treated as surface runoff and the remaining part is treated as groundwater recharge. Runoff from snow melt consists of the snow melt released from the snow storage, which is simulated using a degree-day modelling approach. Besides accumulation and melt, refreezing of snow melt and rain water within the snow storage is included in the model. Gravitational snow transport between grid cells is simulated with the SnowSlide routine [61]. Snow sublimation is estimated using an elevation-dependent potential sublimation function. We assume that the majority of sublimation comes from snowblown sublimation with highest wind speeds prevailing at higher elevations, and therefore potential sublimation is assumed to increase linearly with elevation above 3000 m a.s.l. by a calibrated factor. The actual sublimation is the potential sublimation limited by the snow storage present in the grid cell. Rainfall-runoff consists of the surface runoff from rainfall and lateral flow released from the soil water storage. Soil water processes are simulated for a topsoil and subsoil layer and processes simulated include evapotranspiration, infiltration, percolation, capillary rise, surface runoff and lateral flow. Baseflow is released from the groundwater storage. Each of these four runoff types is routed downstream using a digital elevation model (DEM) and a routing recession function. A detailed description of the model has been published before [51].

**Table 1:** Critical model parameters and their calibrated values.

Parameteracronym	Description	Units	Calibration range	Calibrated value
DDFci^1^	Degree day factor debris-free glaciers	mm °C day^-1^	5–8	7.1
DDFdc^1^	Degree day factor debris-covered glaciers	mm °C day^-1^	2–4.5	3.0
DDFs^2^	Degree day factor snow	mm °C day^-1^	3–6	5.0
SnowSC^2^	Water storage capacity of snow pack	mm mm^-1^	0.2–0.8	0.5
Sm^2^	Minimum slope for gravitational snow transport	m m^-1^	0.01–0.5	0.2
ShdMin^2^	Minimum snow holding depth	mm	0–70	50
SubPot^2^	Potential sublimation function	mm day^-1^	0–0.002	0.0015* (h -3000) for h > 3000 m a.s.l.
αGW^3^	Baseflow recession constant	-	0.001–1.0	0.005
kx^3^	Routing recession coefficient	-	0.5–0.99	0.9476

^1^Calibrated with geodetic mass balance data [73]

^2^Calibrated with MODIS snow cover data [72,81]

^3^Calibrated with observed discharge records (Pakistan Water and Power Development Authority)

### Datasets

**Fig 2:**
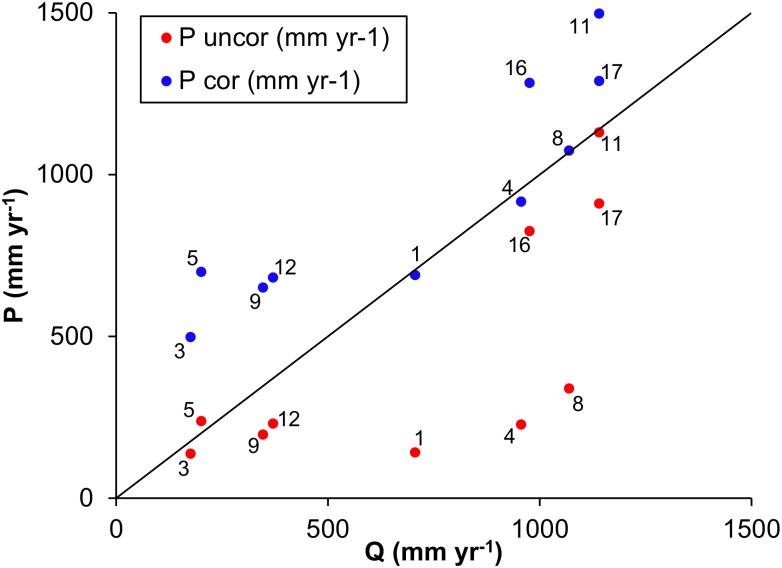
Uncorrected and corrected average annual precipitation plotted versus observed annual average discharge for subbasins listed in Table 2.

Meteorological observations from stations are sparse in the mountains, in particular in the upper Indus. Data mostly originates from valley stations which are not representative for high altitude precipitation. The few stations that are located at higher elevations are typically subject to undercatch in case of snow [62]. Therefore, meteorological datasets consistently seem to underestimate precipitation in the UIB [23,63,64]. As forcing for the SPHY-model we therefore use a corrected precipitation dataset [52], which uses the observation-based APHRODITE [65] dataset as a basis. In the corrected dataset, the raw APHRODITE precipitation data have been corrected by using the glacier mass balance of the major glacier systems as a proxy to estimate high altitude precipitation. Details of the methodology and dataset have been published before [52]. The correction factors that were found in the referred study [52] for 2003–2007 are applied to the daily APHRODITE data for 1971–2000 to generate a 30-year reference climate dataset at 1 km^2^ spatial resolution and daily timestep. By using this dataset we aim to overcome the fundamental problem of underestimated precipitation in distributed modeling of high-mountain hydrology. Because the 2003–2007 period for which the correction factors were derived [52] does not overlap with the 30-year reference period, and we cannot establish the correction factors for earlier periods due to the lack of IceSAT data [66], we validate the correction factors for the 1971–2000 period by comparing the corrected precipitation amounts to observed discharge. We compare average annual precipitation amounts to average annual discharge amounts during periods falling entirely within 1971–2000 (Table 2).Fig 2 shows that the corrected precipitation amounts are in most cases higher than the observed discharge amounts, whereas the uncorrected precipitation amounts are almost all lower than the observed discharge amounts. Because of the systematic underestimation in high altitude precipitation, we conclude that the corrected precipitation dataset is appropriate to be used as historical precipitation forcing in our study.

**Table 2:** Runoff stations used for validation of the corrected precipitation dataset. Catchment areas are delineated based on the SRTM DEM [18]. P_uncor_ is uncorrected APHRODITE [65]. P_cor_ is corrected APHRODITE using published correction factors, which were derived for 2003–2007 [52]. Numbers in parentheses behind station names are for reference in Figs 1 and 2.

Station	Lat	Lon	Area (km^2^)	Observed Q (m^3^s^-1^)	ObservedQ (mm yr^-1^)	P_uncor_(mm yr^-1^)	P_cor_(mm yr^-1^)	Period
Tarbela inflow^a^ (12)	34.329	72.856	203014	2389.2	371	229	681	1977–2000
Mangla inflow^a^ (16)	33.200	73.650	29966	927.9	977	824	1282	1977–2000
Marala inflow^a^ (17)	32.670	74.460	29611	1071.4	1141	909	1288	1977–2000
Dainyor bridge^a^ (1)	35.925	74.372	14147	316.9	706	140	688	1971–1991, 1993–2000
Yogo^b^ (3)	35.183	76.100	64240	359.4	176	136	497	1973–1997
Kharmong^b^ (5)	34.933	76.217	70875	452.3	201	237	698	1982–1997
Doyian^b^ (8)	35.550	74.700	4000	135.7	1070	338	1073	1974–1997
Karora^c^ (11)	34.900	72.767	586	21.2	1141	1129	1496	1975–1996
Shigar^b^ (4)	35.422	75.732	6681	202.6	956	226	915	1985–1997
Shatial Bridge^b^ (9)	35.533	73.567	189263	2083.2	347	195	650	1983–1997

^a^Calculated based on discharge data provided by the Pakistan Water and Power Development Authority (WAPDA).

^b^Based on published data [4].

^c^Based on published data [39].

As digital elevation model (DEM) we use the 15 arc-second void-filled and hydrologically conditioned HydroSHEDS DEM [67], which is based on the SRTM DEM [18]. This DEM is resampled to 1 km^2^ spatial resolution. Glacier outlines are extracted from the Randolph Glacier Inventory [68], and they are recalculated to a fractional glacier cover per 1 km^2^ grid cell. Land use characteristics are extracted from the MERIS Globcover product [69], and quantitative soil properties are derived from the Harmonized World Soil Database (HWSD, [70]) using pedotransfer functions [71].

MODIS snow cover data [72] and geodetic glacier mass balance data [73] are used for model calibration. IceSat derived glacier mass balance data [27] is used for calibration of a basin-scale parameterization of glacier changes [74]. Discharge observations provided by the Pakistan Water and Power Development Authority (WAPDA) are used for model calibration and validation.

### Calibration and Validation

**Fig 3:**
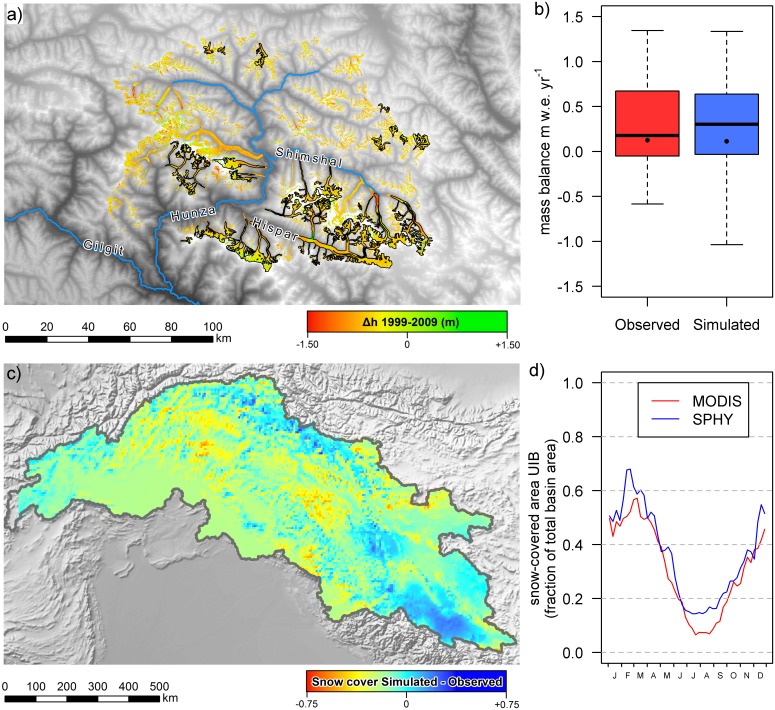
Calibration results for model parameters related to glacier melt and snow melt. a) Elevation difference on glaciers for 1999–2009 derived from DEM-differencing. Outlines of glaciers used for calibration are indicated black. b) Box plots showing the distribution of observed and simulated glacier mass balances for 30 individual glaciers indicated in the area in panel a. Black dots indicate the observed and simulated area-weighted mean mass balance of all considered glaciers. c) Average difference between SPHY simulated snow cover and MODIS observed snow cover. d) MODIS observed and SPHY simulated fractional snow cover averaged over the UIB and averaged for 2000–2007. Source of background data in panel a is the SRTM DEM [18]. Source of background hillshade in panel c is www.naturalearthdata.com.

The model is calibrated using a systematic three-step approach to overcome equifinality problems [75,76]. First, parameters related to glacier melt are calibrated using geodetic mass balance data for the Hunza basin (Figs 1 and 3a). The geodetic mass balance data indicates differences in glacier surface elevation, from differencing SRTM [18] and ASTER [77] DEMs. The SRTM DEM was acquired in February 2000, but due to radar penetration it underestimates glacier elevations and is likely to be more representative of the elevation of glaciers at the end of the 1999 melt season [78]. The ASTER DEMs were collected in late September and early October 2008. The elevation differences are transformed to average annual glacier mass balances (m w.e. yr^-1^) for 30 individual glaciers by using an average ice density of 850 kg m^-3^ [79]. The 30 individual glaciers only include glaciers with a surface area covering at least 5 km^2^ (5 model grid cells) to avoid scale problems, and fractional glacier cover for the individual model grid cells are extracted from an updated version of the ICIMOD glacier inventory, which includes distinction of debris-free and debris-covered ice surfaces for the Hunza basin (courtesy of S.R. Bajracharya). Using the model temperature and precipitation forcing, the glacier mass balances for the individual glaciers are simulated for October 1999 to September 2007. Accumulation is calculated as solid precipitation falling on the grid cells with fractional glacier cover and the adjacent grid cells with a slope steeper than 0.2 towards the glacier surface [52]. The simulated data does not coincide completely with the geodetic mass balance data because the forcing data is only available until 2007. The model parameters related to glacier melt (*DDFci*, *DDFdc*, see Table 1) are then optimized for agreement between the simulated and observed glacier mass balances and different melt parameters are used for debris-covered glaciers and debris-free glaciers. Parameters are optimized by running the model with different combinations of manually sampled parameter values from the parameter ranges listed in Table 1, and the combination of parameters that yields the best agreement between the simulated and observed glacier mass balances is selected.

Second, parameters related to snow storage and melt (*DDFs*, *SnowSC*, *Sm*, *ShdMin*, *SubPot*, see Table 1) are calibrated independently by comparing SPHY simulated snow cover and MODIS remotely sensed snow cover [72]. Remotely sensed snow cover has proven to be useful to improve model calibration in areas with high snow cover [76,80]. The same processed MOD10C2 dataset is used as has been used in another study [81]. From the beginning of 2000 until halfway 2008 the snow cover imagery is averaged for 46 different periods of 8 days (5 days for the last period) to generate 46 different average snow cover maps. That means period 1 is the average snow cover for 1–8 January for 2000 until 2008, period 2 is the average snow cover for 9–16 January for 2000 until 2008, etc. Because the MODIS snow cover product is available at 0.05° x 0.05° spatial resolution, SPHY model snow cover output is averaged over 8 day periods, resampled and projected from 1 km^2^ spatial resolution to the same time periods, resolution and geographic projection as the MODIS product. Parameters related to snow melt are optimised to minimize the difference between SPHY simulated snow cover and MODIS observed snow cover. Parameter values are optimized by running different manually sampled parameter values from the parameter ranges listed in Table 1, and the combination of parameters that yields the smallest difference between simulated and observed snow cover is selected.

Third, after calibration of the model parameters related to glacier melt and snow melt, remaining parameters related to baseflow and routing (*αGW*, *kx*, see Table 1) are calibrated to observed discharge at two locations in the UIB. The selection of locations is primarily dictated by data availability and data access. Secondly the selection is made such that it is a representative subset of the UIB, with different climatic and hydrological characteristics. Calibration is performed at a daily time step for the same periods for which stream flow records are available. Parameter optimization is done using PEST parameter estimation software (freeware, version 6.05) [82]. The calibrated parameters are assumed to be spatially uniform, i.e. one set of parameters is calibrated and assumed to be applicable to the entire UIB.

The calibrated SPHY model is independently validated to observed discharge at two locations that are not used in model calibration.

### GCM downscaling

We select two ensembles containing four GCM runs from the CMIP5 database [83]: one ensemble for the medium stabilization scenario RCP4.5 and one ensemble for the very high radiative forcing scenario RCP8.5. We did not include the mitigation scenario leading to a very low radiative forcing level (RCP2.6). It is unlikely that this RCP can be met, since it requires an immediate drastic decline of emissions followed by ongoing carbon sequestration in the second half of the 21^st^ century, whereas the future emissions expected to come from existing capital are large [84,85]. As we aim to present robust, realistic projections in our study we choose not to include RCP2.6 in the climate model ensemble. By selecting RCP4.5 and RCP8.5 we cover the entire range of radiative forcing resulting from RCP4.5, RCP6 and RCP8.5. To include a wide range of possible futures and because for our study area there is no particular GCM performing best, and no GCM is able to simulate all aspects of the precipitation dynamics in the region satisfactory [42,86,87], we choose to use the entire range of projections available. For both ensembles we therefore select four GCM runs covering the entire spectrum of projected changes in temperature and precipitation, as projected by all the CMIP5 GCM runs with output available for that RCP (Table 3). We select the models closest to the 10^th^ and 90^th^ percentile values of the projections, to avoid the inclusion of outliers, similar as in other studies [3,37,88].

**Table 3:** GCM runs included in the climate model ensemble used to force the hydrological model, and projected changes in temperature and precipitation between 2071–2100 and 1961–1990, averaged over the GCM grid cells covering the upper Indus basin.

RCP	Scenario	GCM	ΔT (°C)	ΔP (%)
**RCP4.5**	DRY, COLD	inmcm4_r1i1p1	2.1	-4.6
	DRY, WARM	IPSL-CM5A-LR_r3i1p1	4.3	-6.3
	WET, COLD	MRI-CGCM3_r1i1p1	2.5	10.5
	WET, WARM	CanESM2_r4i1p1	4.4	13.2
**RCP8.5**	DRY, COLD	MPI-ESM-LR_r1i1p1	6.0	-7.9
	DRY, WARM	IPSL-CM5A-LR_r3i1p1	8.0	-10.2
	WET, COLD	CSIRO-Mk3-6-0_r1i1p1	5.6	29.8
	WET, WARM	MIROC5_r3i1p1	6.7	31.0

The selected GCM runs are statistically downscaled by applying the Advanced Delta Change (ADC) method [89]. ADC has the advantage over the classical delta change method [90,91] that it is not based on changes in the mean, but changes in the entire precipitation distribution, including extreme precipitation, which is a prerequisite for the assessment of changes in hydrological extremes. This is achieved by applying a non-linear transformation to five-day sums of precipitation data. Five-day sums are considered because extreme discharge events usually depend on multiple days of extreme precipitation. The transformation parameters are determined from the GCM control and future runs. Because of the large difference in resolution between the historical dataset (1 km^2^) and the GCM data (∼1.0 to 2.5°), both datasets are interpolated to a common grid of 25 km^2^ resolution. Because ADC focuses on increasing detail in the high end of the precipitation distributions, two different equations are used for the transformation of the observed 5-day precipitation sums, based on the 90% quantile. This quantile is determined per calendar month over the entire reference period for every 25 km^2^ grid cell. The two transformation equations are:

$$P^{*}=aP^{b}\;for\;P^{O}<P_{90}^{O}$$ (2)

$$P^{*}=\bar{E^{F}}/\bar{E^{C}}\;\;\cdot \;\left(P^{O}-P_{90}^{O}\right)+a{\left(P_{90}^{O}\right)}^{b}\;\;for\;\;P^{O}>P_{90}^{O}$$ (3)

Where P* represents the transformed 5-day sums, *P* the reference climate dataset 5-day sums, *P*_*90*_ the 90% quantile and *a* and *b* are the transformation coefficients. The superscripts ^O^, ^C^ and ^F^ denote whether the variable represents respectively the reference climate time series (^O^), the GCM control series (^C^) or the GCM future series (^F^). For 5-day precipitation sums that exceed the *P*_*90*_ of their month an excess value (*E*) is determined: *E* = *P* − *P*_90_. The mean future excess ($\bar{E^{F}}$) and mean control excess ($\bar{E^{C}}$) in Eq 3 are determined per calendar month over the entire future or control period for every 25 km^2^ grid cell:

$$\bar{E^{C}}=\frac{\sum P^{C}-P_{90}^{C}}{n^{C}}\text{ and }\bar{E^{F}}=\frac{\sum P^{F}-P_{90}^{F}}{n^{F}}$$ (4)

The linear scaling of the transformed precipitation with the ratio of future and control excess in Eq 3 expresses a change in the slope of the extreme value plot of the five-day maximum precipitation amounts [89].

The transformation coefficients *a* and *b* are derived from the 60% and 90% quantiles by:

$$b=\;\frac{\text{log}\left\{g_{2}\cdot P_{90}^{F}/\left(g_{1}\cdot P_{60}^{F}\right)\right\}}{\text{log}\left\{g_{2}\cdot P_{90}^{C}/\left(g_{1}\cdot P_{60}^{C}\right)\right\}}$$ (5)

$$a=P_{60}^{F}/{\left(P_{60}^{C}\right)}^{b}\cdot g_{1}^{1-b}$$ (6)

Bias correction factors *g*_*1*_ and *g*_*2*_ account for systematic differences in *P*_*60*_ and *P*_*90*_ in the reference climate time series and GCM control series, and are determined by:

$$g_{1}=P_{60}^{O}/P_{60}^{C}$$ (7)

and

$$g_{2}=P_{90}^{O}/P_{90}^{C}$$ (8)

To reduce sampling variability in the transformation coefficients, the *P*_*60*_ and *P*_*90*_ are smoothed temporally by using a weighted mean with weights of 0.25, 0.5 and 0.25 on respectively the previous, current and next month. The mean excesses are smoothed temporally in a similar manner. A detailed description of the ADC-method has been published before [89].

Because the variability in precipitation within a common grid cell in the UIB is much larger than in the Rhine basin, for which the ADC-method was originally developed, the *a* and *b* parameters are additionally capped to avoid unrealistically high transformed daily precipitation values, which can occur due to the non-linear transformation of the precipitation value. This is done by constraining the *a* parameter and associated *b* parameter as follows:

for b<0.55,  a=6 and b=0.55 (9)

This constraining is based on the distribution of *a* and *b* parameter values observed in the transformation in the Rhine basin [92].

The transformation parameters are determined and five-day sums are transformed for each future period spanning 10 years, by using a moving 30-year window from the GCM future series centered around the 10 year future period under consideration. For example, in the calculation of the transformation parameters for 2061–2070, the GCM future series for 2051–2080 is used. For the last future ten-year period (2091–2100) the GCM future series for 2071–2100 is used, similar as for 2081–2090, because most GCM runs do not go beyond 2100. After transformation a change factor can be determined for each five-day sum, which can be subsequently used to transform the individual days that belong to that specific sum. The change factor R is determined as:

$$R=P^{*}/P$$ (10)

To generate a baseline daily time series spanning 100 years from 2001 to 2100 that can be subjected to the change factors, a series of 100 years of daily precipitation is randomly selected from the 1971–2000 reference climate dataset ($P_{d}^{O}$). The change factor (R) is used to transform the individual daily precipitation sums to future daily precipitation ($P_{d}^{F}$):

$$P_{d}^{F}=P_{d}^{O}\cdot R$$ (11)

Due to the non-linear transformation of precipitation, the mean climate change signal in the bias-corrected downscaled data is modified from the mean climate change signal in the raw GCM data. Such modification of the mean climate change signal is often observed in statistical bias-correction and downscaling methods and may be considered as an undesired deficiency of a bias-correction and downscaling method [93], although this is a current topic of discussion [94–96]. We choose to correct for this effect and therefore the transformed daily precipitation values are scaled for each future ten year period at the grid cell level at monthly scale to the ratio of future and reference precipitation sum according to the raw GCM data as follows:

$$corP_{d}^{F}=P_{d}^{F}\;\cdot \;{\left[\frac{P^{F}}{P^{C}}\cdot \frac{P^{O}}{P_{d}^{F}}\right]}_{m}$$ (12)

With $corP_{d}^{F}$ being the final transformed daily precipitation value, P^F^ being the future precipitation sum in the GCM future run, P^C^ being the precipitation sum in the GCM control run, P^O^ being the precipitation sum in the reference dataset and $P_{d}^{F}$ being the initially transformed precipitation Eq (11).

The temperature transformation, in contrast to that of precipitation, is linear in nature and has the form [89]:

$$T^{*}=\frac{\sigma^{F}}{\sigma^{C}}\left(T-\bar{T^{O}}\right)\;+\;\bar{T^{O}}+\bar{T^{F}}-\bar{T^{C}}$$ (13)

where T* represents the transformed temperature; *T* the temperature in the reference climate dataset; $\bar{T^{O}}$, $\bar{T^{C}}$ and $\bar{T^{F}}$ the monthly mean of respectively the reference, GCM control and GCM future temperature; *σ*^*C*^ and *σ*^*C*^ the standard deviations of the daily GCM control and GCM future temperature calculated per calendar month. The temperature transformation is applied to daily temperature values directly. The same series of 100 years of randomly selected years from the reference period as for precipitation is used for the transformation of air temperature data. The transformation is applied to mean, maximum and minimum air temperature separately (i.e.: *T* in Eq 13 can be replaced by *Tmean*, *Tmax* or *Tmin*).

Each of the downscaled GCM scenarios is used to force the hydrological model with transient runs from 1 January 2001 until 31 December 2100.

### Future glacier changes

Future glacier changes are simulated at large scale for the UIB divided in three sub-regions: one sub-region for each of the mountain ranges Hindu Kush, Karakoram and Himalaya (Fig 1). For each of these three regions a regionalized glacier mass balance model is used to estimate changes in the regional glacier extent as a function of the glacier size distribution in the sub-regions and the downscaled future climate data [74]. This glacier mass balance model is specifically developed for implementation in large-scale hydrological models, where the spatial resolution does not allow for the simulation of individual glaciers and data scarcity is an issue. The model is initially forced with the climatic forcing for the reference period and calibrated to sub-region-averaged glacier mass balance data derived from IceSAT data [27], before it is used to calculate sub-region-scale glacier changes for each of the downscaled GCM ensemble members from 2001 until 2100. The Randolph Glacier Inventory [68] is assumed to be representative for the state of the glacier extent at the start of the future simulation in 2001.

### Future changes in hydrological extremes

Future changes in hydrological extremes are assessed by analysing changes in return levels of extreme discharges with return periods of 2, 5, 10, 20 and 50 years. Return levels are obtained by fitting a Gumbel extreme value distribution [97] through the simulated annual flow maxima during three 30-year periods (1971–2000, 2021–2050 and 2071–2100).

## Results and Discussion

### Calibration and validation

After calibration of the degree-day factors of debris-covered and debris-free glaciers (Table 1) the area-weighted mean glacier mass balance (+0.11 m we yr^-1^) matches very well with the observed area-weighted mean glacier mass balance (+0.12 m we yr^-1^) (Fig 3b). The interquartile range is also similar. However, the total spread within the sample of 30 individual glaciers is larger in the simulation than in the observations. The larger spread in the simulation stems most probably from the quite coarse model resolution at 1 km^2^. The calibrated values for the degree day factors for debris-free and debris-covered glaciers (Table 1) fall well within the range of values derived in field experiments in the greater Hindu-Kush-Himalayan region [98]. Given the large scale and the fact that we use a fixed parameter set for all glaciers we conclude that the calibrated parameters can be considered representative for the UIB.

Averaged over the UIB, the calibrated SPHY model simulates snow cover reasonably well (Fig 3c and 3d). The largest overestimates occur in the Karakoram range and the Himalayan range in the most southeastern part of the UIB. The largest underestimates occur in the Hindu Kush and mountain ranges to the south of the Karakoram. At the basin scale, there is also a slight overestimation of snow cover during most parts of the year (Fig 3d). Overestimates may well be related to the fact that snow redistribution by wind from one grid cell to another is not included in the SPHY model. Another explanation could be related to the simple approach used to estimate sublimation, whereas sublimation can potentially be an important component of the high-altitude water balance in the HKH region [99]. Studies in other areas revealed that blowing snow sublimation plays a larger role than ground sublimation from the snow pack, and that sublimation losses can be in the order of tens of percents of the total snow accumulation, and up to ∼90% on very windy ridges [100–102].

**Fig 4:**
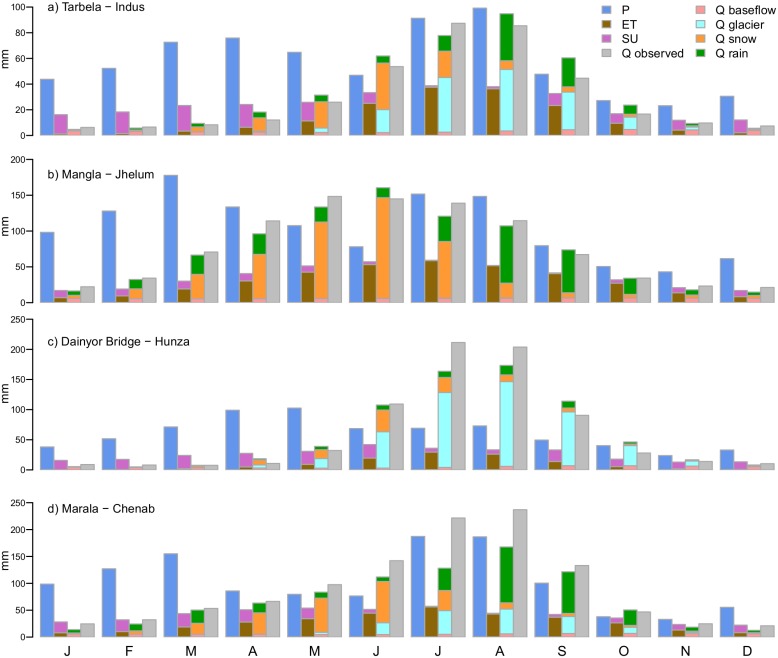
Monthly-averages of most important water balance terms and observed discharge for catchments used for calibration and validation (Table 4). Plots show precipitation (P), evapotranspiration (ET), sublimation (SU), observed discharge (Q observed), baseflow (Q baseflow), glacier melt (Q glacier), snow melt (Q snow) and rainfall-runoff (Q rain).

Calibration to observed discharge shows that averaged over the two locations, the Nash-Sutcliffe efficiency [103] calculated at a daily time step equals 0.81, whereas Pearson’s correlation coefficient equals 0.92 (Table 4). For the location at Tarbela, covering a large part of the Indus basin, there is a positive bias of 9.7% in the simulation. The bias is largest during the months with high contribution of snow melt to the discharge (Fig 4a), and is thus likely related to the overestimate of snow cover on the part of the Tibetan Plateau that is part of this catchment (Fig 3d), which in turn relates to the high precipitation forcing in spring. For the Jhelum basin upstream of Mangla reservoir, with a large contribution of snow melt to the stream flow, the model simulates the seasonal patterns in stream flow well (Fig 4b). For the Hunza basin upstream of Dainyor bridge, which harbours the highest and most scarcely monitored part of the UIB and is used for model validation, simulated stream flow is slightly underestimated during the peak season in July and August, and overestimated during September and October (Fig 4c). During these months the stream flow is dominated by glacier melt, which is driven by air temperature. This suggests that the APHRODITE temperature fields may lack some accuracy for this area where observations are very scarce. Besides the model slightly underestimates snow cover in the northern part of the Hunza basin (Fig 3c), which may contribute to the underestimate of the flow peak. For the Chenab basin, located to the southeast of the Jhelum basin, the model underestimates the flow during July and August, leading to a large negative bias (Fig 4d). In this case the bias is most likely related to a shortage of precipitation in the forcing data, being 1222 mm yr^-1^ for the validation period whereas the observed discharge is only slightly lower (1100 mm yr^-1^).

**Table 4:** Correlation of observed and simulated discharge at locations used for model calibration and validation. Correlations coefficients were calculated at a monthly time step.

ID in Fig 1	Name	River	Calibration (C) or Validation (V)	Used period (observation interval)	Nash-Sutcliffe efficiency (-)	Pearson’s coefficient of correlation (-)	Bias (%)
12	Tarbela Inflow	Indus	C	Apr 1976-Dec 2007 (10 days)	0.78	0.91	9.7%
16	Mangla inflow	Jhelum	C	Apr 1976 –Dec 2007 (10 days)	0.84	0.93	-6.4%
1	Dainyor Bridge	Hunza	V	1966–2004 (daily)	0.76	0.88	-2.8%
17	Marala Inflow	Chenab	V	Apr 1976-Dec 2007 (10 days)	0.71	0.90	-23.1%

The annual water balance for 2003–2007, largely coinciding with the period covered by IceSAT derived glacier mass balances for three sub-zones in the UIB (Fig 1), is plausible for the Indus upstream of Besham Qila with precipitation input being 664 mm yr^-1^, the negative glacier mass balance contributing 25 mm yr^-1^, and evapotranspiration, sublimation and discharge being 174 mm yr^-1^, 139 mm yr^-1^ and 367 mm yr^-1^, respectively on the other side of the water balance. The gap in the water balance of 9 mm yr^-1^ is negligible and can be attributed to changes in storages in the soil, snow cover and groundwater. Given the complexity of high mountain hydrology, the scale of the application, the use of one parameter set for the entire basin, and large uncertainties in the meteorological model forcing, we conclude that the model performance is satisfactory for our purpose to estimate the impacts of climate change for the UIB’s future hydrology.

### Present day hydrology

**Fig 5:**
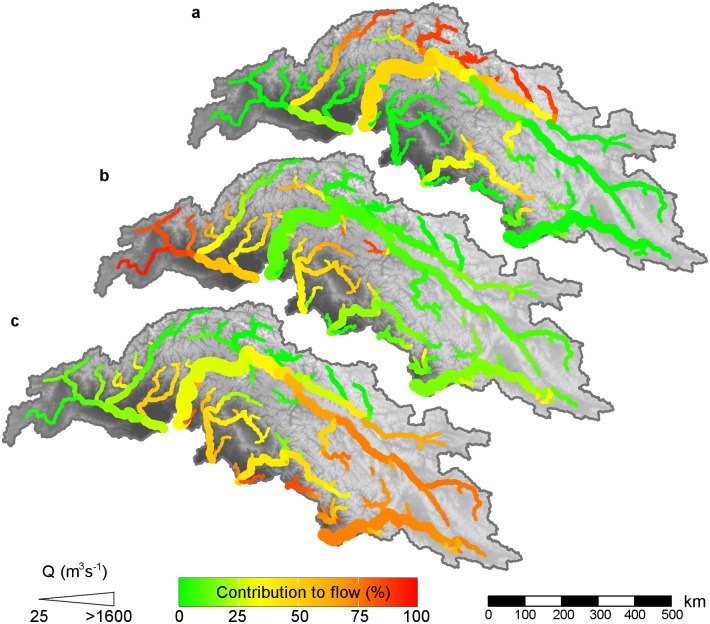
Streamflow composition in the upper Indus basin. Contributions of glacier melt (a), snow melt (b) and rainfall-runoff (c) to the total flow averaged over the reference period (1971–2000). The magnitude of streamflow is indicated by the symbol size. Source of background data is the SRTM DEM [18].

The stream flow compositions during the reference period have a large spatial variation in the UIB (Fig 5). Strong south to north and east to west gradients are visible in the intensity of the rainfall-runoff generation, consistent with the intensity of the monsoon that comes in from the southeast during the monsoon season. In the monsoon-dominated Sutlej basin the contribution of rainfall to the total flow at the outlet is 74%, whereas this is 33% for the Indus at Tarbela. Snow melt has highest importance in the water coming from the Hindu Kush mountains in the Kabul basin, which receive large amounts of solid precipitation from westerly disturbances during the winter months. Glacier melt contribution is highest in the most glaciated Karakoram subbasins, like Hunza (85%) and Shigar (43%), and the upstream reaches of Kunar. This makes the Indus river the most melt-water dependent river leaving the UIB (55% glacier- and snow melt at Tarbela). The lower-latitude Satluj, Beas, Ravi, Chenab and Jhelum rivers are dominated by input from rainfall, most of which falls during the monsoon season. The Jhelum river also has a substantial snow melt component (32%). Our estimates of stream flow composition match reasonably well with what others found based on a conceptual model [4,5]. The results from this study show a slight shift in runoff composition towards higher contribution of snow melt and rainfall and lower contribution of glacier melt compared to our earlier findings [3]. This is because the current study is focused on the UIB only whereas the earlier study comprised the upstream basins of five Asian rivers, and most importantly because in the current study we use precipitation forcing that is corrected for the underestimate of high altitude precipitation, whereas this was not available at the time of the 2014 study. In the 2014 results, the shortage of precipitation input is compensated by higher glacier melt rates when calibrated only to observed stream flow, a common problem in the simulation of mountain hydrology [76]. Associated to the large differences in the stream flow composition between the tributaries are also differences in the intra-annual distribution of river discharge. Although the peak of glacier melt largely coincides with the peak in monsoonal rains, the snow melt peak occurs during spring. These contrasts in hydrological regimes and stream flow composition of the different tributaries feeding the downstream basin may lead to different responses to future climate change.

### Future climate

**Fig 6:**
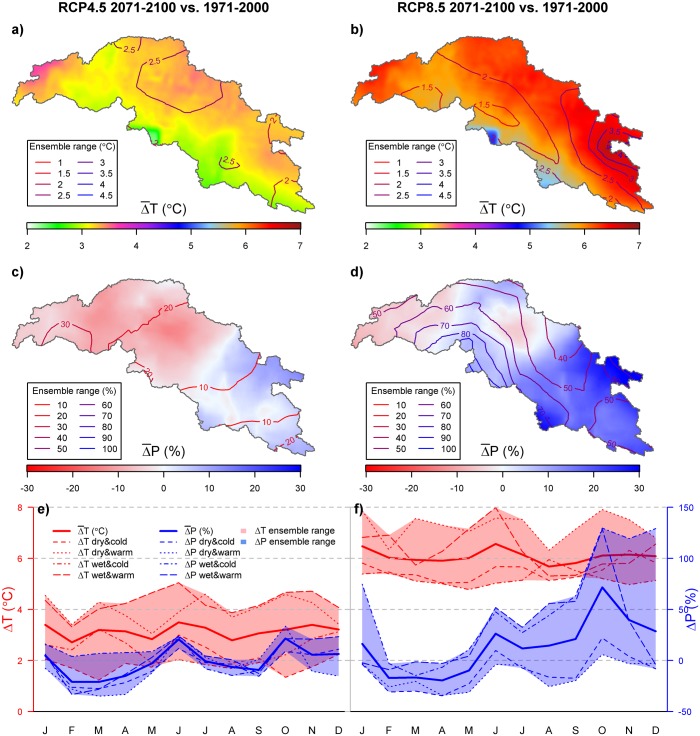
Projected changes in temperature and precipitation for 2071–2100 compared to 1971–2000. Projections are shown for the RCP4.5 (left panels) and RCP8.5 ensembles (right panels). a,b) Ensemble mean change in air temperature. Contour lines denote the ensemble range of projections. c,d) Ensemble mean change in annual precipitation sum. Contour lines denote the ensemble range of projections. e,f) Ensemble mean changes in air temperature and precipitation per month of the year. Shading denotes the ensemble range of projections.

The downscaled GCM ensembles for RCP4.5 and RCP8.5 show that the future climate in the UIB is highly uncertain. Both ensembles indicate strong warming (Fig 6a and 6b), with significantly stronger warming for the parts of the basin with the highest elevation. The difference in warming can be up to ∼1°C (RCP4.5) and ∼2°C (RCP8.5) between the lowest and the highest areas in the UIB. This is well in line with presently observed elevation-dependent warming [104,105]. Comparing the average warming in the UIB (+2.1 to +8.0°C between 1971–2000 and 2071–2100) to the global average (+1.8 to +4.4 between the same periods for the same RCPs [106]), also demonstrates that the UIB is likely to warm stronger than other parts in the world. The uncertainty in warming is largest in the eastern and northern parts of the UIB. Seasonal differences in the temperature projections are limited. For both RCPs, strongest temperature increases are projected for January and June (Fig 6e and 6f). These projected temperature changes are well in line with what was found in other studies for the Indus basin [44,45], although the different scenarios and climate models used in those studies make a direct comparison difficult.

The precipitation projections are highly uncertain. The RCP4.5 mean projection shows clear contrasting trends of precipitation increase in the southeastern part and precipitation decrease in the northwestern part of the UIB (Fig 6c). This contrast is also observed for the RCP8.5 mean projection (Fig 6d), although the area with a projected increase in precipitation is larger. Besides, the precipitation increase is much higher for RCP8.5 than for RCP4.5, and the magnitude of precipitation decrease is smaller. The range of the precipitation projections however is very large. For both ensembles, for each geographical location there are both ensemble members that predict an increase and a decrease in precipitation. The ensemble range is much larger for RCP8.5 compared to RCP4.5 and can be up to 100% for the most downstream parts. The mean projected precipitation trend in the southeastern parts of the UIB suggest that monsoon intensity increases, and that the monsoon protrudes further to the northwest with increasing temperatures. Averaged over the UIB, significant seasonal patterns can be observed in the precipitation projections (Fig 6e and 6f). In general, although subject to a large uncertainty, the mean projection in both ensembles is indicating precipitation decrease during February-May and increase in October-January, with the increase being strongest in October. Especially the months October through January have very large uncertainties in RCP8.5. Despite this large uncertainty, an increase in precipitation is likely. Shifts in precipitation patterns originating from westerly disturbances are more difficult to interpret. The increasing precipitation during winter months combined with decreases during the early spring months could suggest that the westerly disturbances set in earlier, however the spatial pattern reveals mostly a precipitation decrease (on annual scale) in those areas where westerly disturbances are the main contributor to total precipitation. The trends, and large uncertainties of precipitation change we find in our ensembles are similar to what was found in an analysis of 32 CMIP5 GCMs over the Hindu-Kush-Karakoram-Himalaya region [42], and once more demonstrate the need for improvement of climate simulations in this region, to lower the uncertainty in the future’s climate.

### Future glacier extent

The large uncertainty in the climate change scenarios translates in the projected changes in glacier extent (Table 5). Even though the wet scenarios project large increases in precipitation, glacier area decreases considerably during the 21st century throughout the basin, since the precipitation increases cannot compensate for the ample rises in temperature. Our projections are in the same order as projections made in recent other studies at large scale [107,108].

**Table 5:** Projected remaining glacier area (%) in 2100 compared to the reference situation for three sub-regions in the upper Indus basin, when forced by the individual ensemble members.

RCP	Scenario	GCM run	Remaining glacier area (%) in 2100 compared to RGI		
			Himalaya	Hindu Kush	Karakoram
RCP4.5	DRY, COLD	inmcm4_r1i1p1	34.6	29.5	50.3
	DRY, WARM	IPSL-CM5A-LR_r3i1p1	17.7	12.3	27.1
	WET, COLD	MRI-CGCM3_r1i1p1	41.6	54.5	64.9
	WET, WARM	CanESM2_r4i1p1	15.4	8.0	26.1
RCP8.5	DRY, COLD	MPI-ESM-LR_r1i1p1	13.8	12.6	28.6
	DRY, WARM	IPSL-CM5A-LR_r3i1p1	9.5	7.7	13.6
	WET, COLD	CSIRO-Mk3-6-0_r1i1p1	15.2	12.9	30.9
	WET, WARM	MIROC5_r3i1p1	12.4	6.4	14.0

### Future hydrology

The uncertainty in UIB’s future climate evidently also reflects in the projections of the future hydrology. Nevertheless, several remarkably consistent patterns of projected hydrological changes can be observed across the range of scenarios.

#### Stream flow composition

**Fig 7:**
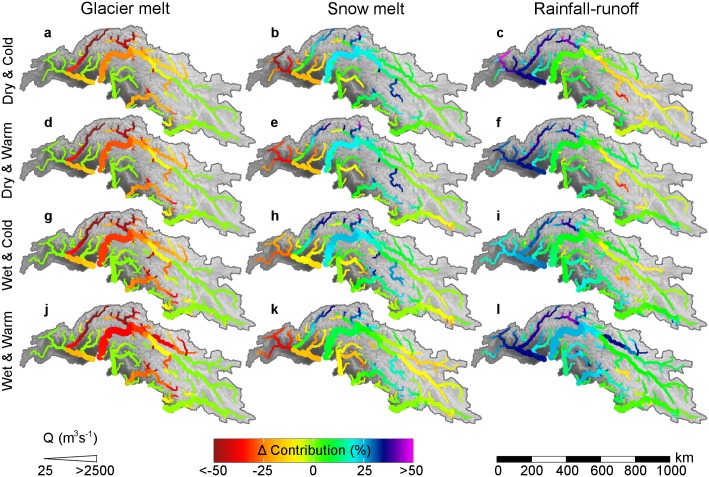
Changes in the contributions of individual components to the total flow for 2071–2100 compared to 1971–2000 for the cryospheric-hydrological model forced by the downscaled GCMs in the RCP8.5 ensemble. Changes are calculated as the contribution to stream flow in the future period (%) minus the contribution to stream flow in the reference period (%). Source of background data is the SRTM DEM [18].

The contribution of glacier melt is projected to decrease by the end of the century across all scenarios (Fig 7a, 7d, 7g and 7j). For RCP8.5 the decrease is strongest for the wet, warm scenario and smallest for the dry, cold scenario. The changes in snow melt contribution also show a consistent signal across scenarios, but with high spatial variation (Fig 7b, 7e, 7h and 7k). The strongest decreases are projected for the Hindu Kush mountain range consistent with the high warming rates. In the Karakoram and in the Zanskar subbasin, the contribution of snow melt increases in favor of glacier melt, since the glacier area is reduced but seasonal snow still provides a considerable amount of melt water. Although the strongest precipitation increases are projected for the winter months (Fig 6e and 6f), all year increases in temperature lead to a shift in the precipitation regime to more precipitation falling as rain instead of snow. For the ensemble means, averaged over the UIB the portion of the precipitation falling as rain changes from 58% during 1971–2000 to 66% during 2071–2100 for RCP4.5 and 75% for RCP8.5, consistent with earlier projections of changes in UIB snowfall [109]. Remarkably, despite this shift in precipitation regime, snow melt contribution to total runoff increases or stays equal in most parts of the UIB except for the Kabul basin (Fig 6b, 6e and 6h). This can be explained by the combined effect of increased evapotranspiration due to higher temperatures and increased water availability in the soil and a reduction of sublimation due to decreases in snow cover. For the western part of the Kabul basin the strongest increases in temperature are projected (Fig 6a and 6b), leading to a reduction in snow melt contribution and increase in rainfall-runoff contribution across scenarios. The RCP8.5 wet & warm scenario leads to largest increases in rainfall-runoff contribution (Fig 7l) and for this scenario the contribution of snow melt is mostly reduced (Fig 7k).

#### Water availability and intra-annual shifts

**Fig 8:**
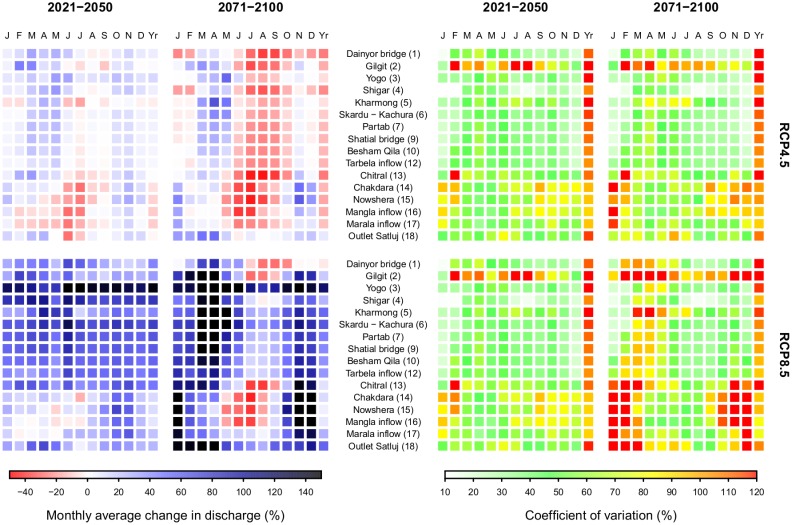
Ensemble mean monthly average changes in discharge at individual locations in the upper Indus basin (left) and coefficient of variation for the entire ensemble (right). Changes are shown for the near future (2021–2050) and the far future (2071–2100) compared to 1971–2000, for the RCP4.5 and RCP8.5 ensembles. Numbers in parentheses behind the location names refer to the locations in Fig 1.

Changes in stream flow composition are also related to hydrological changes in different times of the year (Fig 8). For most catchments (1–13) in the near future (2021–2050) for RCP4.5, flows show little changes during the high flow season and increase during autumn and especially spring. This is most likely due to an increase in autumn and winter precipitation (Fig 6e and 6f) and earlier onset of snow- and glacier melt. Despite the projected decrease in annual precipitation in most of the main Indus branch’s basin (Fig 6c), annual-averaged water availability is unchanged for the locations in the Indus river (6–12), and increases slightly for the upstream subbasins of Hunza, Shigar and Shyok. For Hunza and Shigar this is most likely related to increased glacier melt, and for the Shyok basin upstream of Yogo it is a combination of precipitation increases for the mean of the scenarios (Fig 6c) and increased glacier melt. The lower altitude subbasins (14–17) show a different pattern of seasonal shifts, with strong decreases in flow during June and July and often also for the spring months. Autumn and winter flows increase slightly, and annual-averaged water availability decreases slightly for these sub-basins. These basins have large rainfall-runoff and snow melt components, and decreases in precipitation during spring and the monsoon season combined with higher evapotranspiration rates, most likely cause runoff to decrease during those months, whereas precipitation increases during the winter months, cause increasing runoff during winter (Fig 6e). For the end of the century (2071–2100), the mean projection for RCP4.5 shows similar changes in intra-annual water distribution as for the near future, but much more pronounced. As glacier areas have reduced significantly by then, the amount of glacier melt water decreases substantially, causing reductions in discharge during the summer months. In addition, flows in the high flow season decline further by reduced precipitation during the monsoon season, and total water availability decreases for the entire UIB due to reduced precipitation in combination with increased evapotranspiration. Flows in spring tend to increase more strongly due to earlier onset of snow and glacier melt during these months. Only for the Satluj river, being the most rain-dominated river in the UIB, increases in water availability are projected for the far future according to the RCP4.5 ensemble mean since precipitation is projected to increase for this part of the UIB (Fig 6c).

In terms of total water availability, the RCP8.5 ensemble shows quite contrasting projections with increases in annual water availability in the near and far future. The patterns in the shifts in the ensemble mean projection however are consistent with RCP4.5, implying a transition to a more attenuated hydrograph. That flows increase during all parts of the year, including the high flow season, is most likely because precipitation is projected to increase for all seasons except spring and glacier melt rates in the near future increase stronger compared to RCP4.5 due to stronger temperature increase. Earlier onset of melt in spring causes runoff to increase during spring despite reduced precipitation input during this season. The projection for the far future shows that despite strong precipitation increases, the glacier-melt dominated Chitral, Hunza, Gilgit and Shigar subbasins experience reductions in flow during the high flow season, since the glacier extent has decreased strongly by then (Table 5). The similar contrasting shifts between the high-altitude and lower altitude subbasins as for RCP4.5 can be observed. Besides, the contrast in the precipitation projections between the Kabul subbasin and the remaining part of the UIB (Fig 6d) are also visible in the projections of changes in total water availability. The remarkable strong year-round increase in flows in the near future as well as far future for the Shyok subbasin, can most likely be explained by the fact that projected precipitation increases are strongest in this subbasin (Fig 6d). Similarly strong year-round increases in flow for the rain-dominated Satluj river can be explained by strong precipitation increases in this subbasin.

Our results of intra-annual changes are in line with the projections made for the Shigar catchment [38]. There, the initial increase of summer flows is projected halfway through the century followed by a decline at the end of the century, that is accompanied by increasing flows in spring. A previous study projects increasing flow in the UIB until the end of the 21^st^ century, with more rapid increase during the first half of the century [45]. The authors assessed changes for RCP4.5 and RCP8.5, and used one downscaled GCM and one RCM for their projections. They project stronger increase in winter flows compared to summer flows, consistent with our results. Similar results were found using the previous generation IPCC scenarios A2 and B2 for one RCM [110]. Accurate comparisons to the cited studies is however hampered by the use of different scenarios and climate models.

The patterns are consistent for both RCPs, but the uncertainty is large: for the combined RCP4.5 and RCP8.5 ensembles total water supply from the UIB in 2071–2100 changes by -15% to +60% with respect to 1971–2000. Large uncertainties in hydrological projections have also been found earlier for the Shigar catchment [37,38], and at larger scale [111]. Striking is the particularly large uncertainty observed for the Gilgit subbasin in both RCPs (Fig 8), which is most likely caused by a particularly large uncertainty in monsoon and autumn precipitation and summer air temperatures in both RCPs for this subbasin.

#### Hydrological extremes

**Fig 9:**
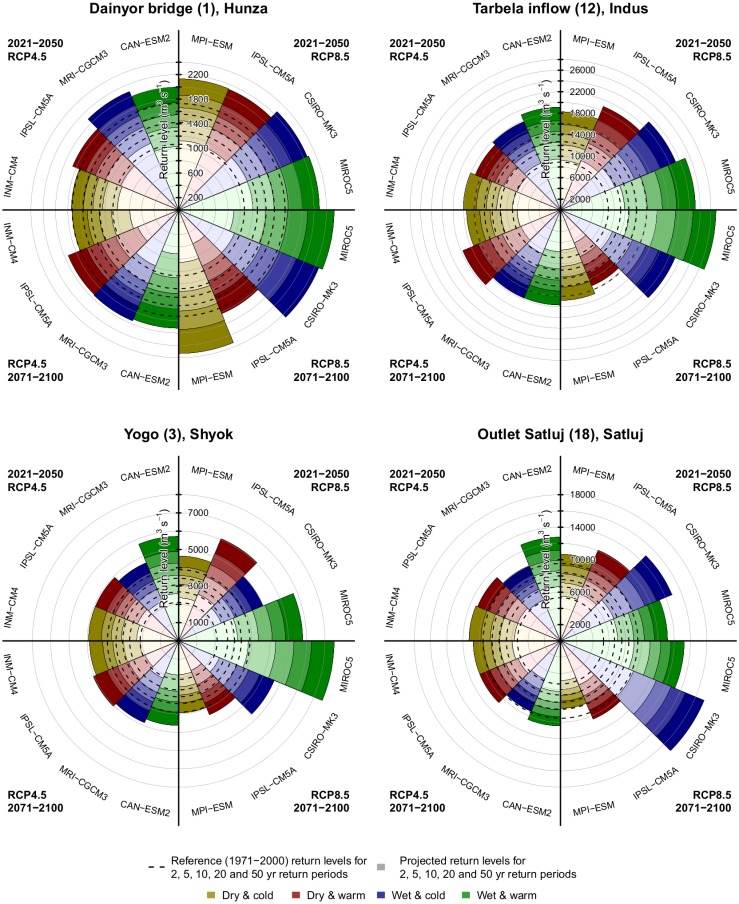
Return levels at four locations in the UIB for 2, 5, 10, 20, 50 years return periods for the hydrological model forced with the individual downscaled GCMs, for the near future (2021–2050) and the far future (2071–2100). Dashed lines indicate the corresponding return levels during the reference period (1971–2000). Each plot represents one location in the UIB. Dashed lines indicate the corresponding return levels during the reference period (1971–2000). Projected return levels are shown for individual downscaled GCMs for RCP4.5 near future (upper left quarter), RCP4.5 far future (lower left quarter), RCP8.5 near future (upper right quarter), and RCP8.5 far future (lower right quarter). Projected return levels for different return periods are incrementally indicated by the color intensity of the different layers in each of the slices.

Large changes in extreme discharges can be expected for most parts of the UIB (Fig 9). For most rivers, the highest water levels occur during the coinciding melting and monsoon season, and therefore the changes in return levels are largely determined by the projected climate changes during those months. However, the peak flows are also significantly determined by the meltwater from snow and glacier melt stemming from winter precipitation, forming a basic flow level during the melting and monsoon season which is exacerbated by runoff originating from extreme precipitation events.

Remarkably, the return levels for extreme discharges in the very upstream Hunza river with its highly glaciated basin increase for all scenarios, including the scenarios projecting overall dryer conditions. For the Hunza river with a large contribution of glacier melt, the decreases in glacier extent play a large role, lowering the continuous flow from glacier melt during the melting season. Nevertheless, even for the far future, when the contribution of glacier melt and the total flow has significantly decreased, the extremes in discharge are clearly increasing, due to increases in extreme precipitation, across scenarios. Earlier work in the Shigar subbasin to the east of Hunza also indicates that hydrological extremes may considerably increase until the end of the century [38,49].

The return levels for extreme discharges at Tarbela, where the main Indus branch leaves the UIB, increase for most scenarios as well, except the RCP8.5 dry & warm scenario, because precipitation events are projected to be more intense across the climate model ensemble. At Tarbela, the most extreme changes in return levels are projected for the RCP8.5 wet & warm scenario, with 100-years return level increasing by more than 100% between 1971–2000 and 2071–2100.

For the Shyok basin, return levels clearly increase most for the RCP8.5 wet & warm scenario, which also project the largest precipitation increases. Interestingly, the RCP4.5 wet & warm scenario projects stronger return level increases for the near future compared to the far future, despite increasing precipitation intensity. Since the Shyok river has a large glacier melt contribution, this is related to the lower continuous flow from glacier melt during the melting season in the far future.

At the outlet of the rainfall-runoff dominated Satluj basin the range of projected changes in return levels is largest. As this is the most rainfall-runoff dominated river, the discharge of this river is also most sensitive to changes in extreme precipitation. In addition, precipitation projections have large spread for this part of the UIB (Fig 6c and 6d), which may also imply a large spread in the projected precipitation extremes.

The model runs forced with the RCP8.5 MIROC5 and CSIRO-Mk3 GCMs clearly stand out from the other model runs for Satluj, Shyok, and the Indus at Tarbela, projecting the wettest future and strongest increases in precipitation intensity. Since the uncertainty in future climate is larger for the RCP8.5 ensemble compared to the RCP4.5 ensemble, it is not surprising that the range of the projected return levels is also larger for the RCP8.5 ensemble.

### Uncertainty

This study sheds light on the propagation of uncertainty in the future climate for the future hydrology. We emphasize that the future climate in the upper Indus basin is highly uncertain as none of the current state-of-the art GCMs and RCMs satisfactory simulates the monsoon and westerly dynamics in the region [46,86,112], making the reliability of future scenarios questionable. We stress the importance of improvement in the representation of the complex climate in High Mountain Asia in order to be able to narrow down the uncertainty in future projections.

The ensemble of selected climate models determines the outcome of a climate change impact study to a large extent. In this study, climate models to be included in the climate model ensemble have been selected based on the envelope of projections of mean air temperature and precipitation totals. Other selection approaches, based on climate model skill [e.g. 113], changes in multiple climate properties [e.g. 114,115], or a combination of envelope and skill [e.g. 15] will lead to different climate model ensembles and thus to different projections of climate change and its impacts.

There are many different statistical downscaling approaches and choosing the most appropriate method is challenging, especially for areas with complex climate and terrain like the UIB. A study comparing different empirical-statistical downscaling methods for precipitation in the Austrian Alps concludes that methods which apply a non-linear transformation, like ADC does, are among the best performing approaches over terrain with complex topography [116]. A successful application of a method [117], on which ADC is based, has been reported for mountainous parts of the Rhone basin [118]. Although the ADC method was not specifically developed for application in the Hindu Kush-Himalayan region and we could not validate its performance this does provide confidence in its applicability. As in any study projecting future changes in extremes, the uncertainty of the downscaled projections increases with the return period of the events, because these only occupy a very small part of the precipitation intensity distribution. In addition, the ADC method focuses on proper transformation of multi-day precipitation events, assuming these to be the main driver of flooding events. Although we believe that multi-day precipitation events are also the main driver of flooding events in the UIB, future studies should also pay particular attention to changes in multi-day events of high air temperature during the melting season, as these are probably also important drivers of flooding events in the upstream parts of the UIB. In that respect it would be recommendable to test the application of the Quantile Mapping [e.g. 119] downscaling methodology to climate change projections for the UIB, which correct each quantile of the precipitation and air temperature distributions separately and have demonstrated good performance over terrain with complex topography [37,116].

Besides the uncertainty within climate model ensembles, climate models themselves, and empirical-statistical downscaling, additional uncertainties are introduced in the hydrological model forcing and other data, parameters, and representation of physical processes. Precipitation products show a large range of precipitation amounts for High Mountain Asia [23]. Although we use climate model forcing that is corrected for the underestimate of high-altitude precipitation [52], these data can still have large biases. For example, the UIB-averaged corrected precipitation is estimated to be 913±323 mm yr^-1^ between 2003 and 2007. Another study assessed precipitation in the UIB based on station data and precipitation estimates for the accumulation zones of major glaciers [64]. The study found similar precipitation totals as reported in the dataset which we used for our study [52], providing further confidence in the used precipitation input. Further narrowing down of the uncertainty in historical precipitation data is a prerequisite for better estimates of future climate change impacts [120]. Similarly, air temperature datasets show a large spread over the UIB. An analysis of the sensitivity of glacier melt water amounts to the large variation in baseline air temperature data shows that variation can lead to an estimated glacier melt contribution to total flow varying from 4 to 78% for the baseline climate, and even larger variations in future projections [121]. Important data used in this study that also introduce uncertainty are the subregion-averaged glacier mass balance data derived from IceSAT data [27], since they are used for the calibration of the large scale glacier change parameterization.

Uncertainties are introduced by using a uniform set of calibrated model parameters for the entire domain. The values of most of the calibrated parameters vary in space and time in reality. However, due to the lack of data which can be used for calibration that covers the entire UIB, we are limited to calibrate the parameters for different subareas and limited temporal periods of our model domain and extrapolate them to larger areas and time periods. This is a common problem in model calibration for data-scarce areas like the UIB [76]. The increasing availability of geodetic glacier mass balance data can help to calibrate spatial variation in degree-day factors for glacier melt, and this can potentially also be done for degree-day factors of snow using MODIS snow cover data. The calibration of parameters which are calibrated using observed discharge are limited to the subbasins which have discharge data available. Different sets of parameters could have been calibrated for each of the gauged subbasins, but then difficulties would arise in assigning parameter values to the ungauged part of the basin. This could in the future potentially be (partly) overcome by categorizing subcatchments by different characteristics, such as climatic differences, degree of glaciation and catchment size. Parameters could then be calibrated for gauged catchments and transferred to ungauged catchments. The potential of such approaches [122] in the UIB region are to be explored in the future.

Parameters themselves have their own uncertainties, which are ideally all taken into account. A study compared three sources of model uncertainty (model parameters, climate projections, natural climate variability) for future projections for a hydrological modeling study in the Hunza subbasin [123]. The study showed that, for heavily glacierized basins, the uncertainty stemming from parameter uncertainty often exceeds the uncertainty stemming from uncertainty in the future climate and natural climate variability. In the cited study an ensemble of three GCMs was used. When a larger ensemble of climate models with a wide range of projections is used, like in our study, the uncertainty stemming from uncertainty in the future climate is probably larger. By calibrating model parameters in a three-step approach using geodetic glacier mass balance data, snow cover data, and observed discharge, the parameter uncertainty is reduced, because the model parameters are constrained to the processes they are affecting, reducing equifinality problems [76].

Uncertainties in the mapping of glacier extent have implications for the simulated contribution of glacier melt to the total flow. For the Karakoram range, the total glacier area according to three different glacier inventories varies from 21193 to 26018 km^2^ (i.e. the highest estimate is ∼23% higher than the lowest estimate) [124], and thus the inventory used will have large consequences for the simulated amounts of glacier melt during the reference period and may also lead to different simulated hydrological responses to climate change. The glacier inventory also determines the initial glacier volume, which is used as a starting point for the simulation of glacier change projections, which is based on volume-area scaling in our approach [74,125]. Besides, the method which is used to estimate initial ice volume from glacier outlines can also vary. A comparison of six different methods to calculate ice volume showed ice volume in the Karakoram ranging from 1683 to 2827 km2 (i.e. the highest estimate being ∼68% larger than the lowest estimate) [126]. More uncertainties are involved in the glacier change projections as discussed in earlier published work [74]. Currently no basin-wide map with distinction of debris-free and debris-covered glaciers is available for the UIB, and thus the differentiation of both glacier surface types is based on assumptions of elevation and slope constraints controlling the glacier surface type. A map with distinction of both glacier surface types would solve this key issue. Another key issue is the limited understanding of the role of sublimation in the high mountain water balance [99,101].

## Conclusions

In this study we use a distributed hydrological model which we force with the latest suite of climate models using an advanced statistical downscaling technique. This study stands out from previous work as for the first time shifts in seasonal water availability are assessed in combination with changes in hydrological extremes at basin scale for the upper Indus basin.

Assessing future hydrological changes in the upper Indus basin is complicated by large uncertainties in the historical and future climate, uncertainties in glacier extent and glacier mass balance, and uncertainties in hydrological model processes and parameters.

From our results we can conclude that the upper Indus basin faces a very uncertain future in terms of water availability in the long run. Projections of changes in water availability from the upper Indus basin at the end of the 21^st^ century range from -15% to +60% with respect to 1971–2000. This uncertainty mainly stems from the large spread in the projections of precipitation change throughout the 21^st^ century. Therefore, formulating adequate adaptation measures which take into account the uncertain future is of vital importance, thus requiring hydrological projections to be made based on an ensemble of climate models representing all possible futures.

Despite the large uncertainties in future climate and water availability, basin-wide patterns and trends of intra-annual shifts in water availability are consistent across climate change scenarios. These trends mainly consist of minor increases in summer flows combined with increased flows during other seasons in the near future (2021–2050) and decreases in summer flows combined with stronger increasing flows during the other seasons in the far future (2071–2100). Furthermore, increases in intensity and frequency of extreme discharges are found for most of the UIB and for most scenarios and models considered, implying increases in flooding events during the 21^st^ century.

Population growth in combination with increasing standards of living and associated increases in energy and food production will continue to expand the downstream water and energy demand [127,128]. This implies a growing dependency on the uncertain future water resources, which calls for sound basin-wide adaptation strategies to be developed across sectors that take into account the changing demand and supply in the Indus basin as well as uncertainties therein.

## References

[1] Immerzeel WW, Bierkens MFP. Asia’s water balance. Nat Geosci. 2012;5: 841–842. 10.1038/ngeo1643

[2] Immerzeel WW, Van Beek LP., Bierkens MFP. Climate change will affect the Asian water towers. Science. 2010;328: 1382–5. 10.1126/science.1183188 20538947

[3] Lutz AF, Immerzeel WW, Shrestha AB, Bierkens MFP. Consistent increase in High Asia’s runoff due to increasing glacier melt and precipitation. Nat Clim Chang. 2014;4: 587–592. 10.1038/NCLIMATE2237

[4] Mukhopadhyay B, Khan A. A quantitative assessment of the genetic sources of the hydrologic flow regimes in Upper Indus Basin and its significance in a changing climate. J Hydrol. Elsevier B.V.; 2014;509: 549–572. 10.1016/j.jhydrol.2013.11.059

[5] Mukhopadhyay B, Khan A. A reevaluation of the snowmelt and glacial melt in river flows within Upper Indus Basin and its significance in a changing climate. J Hydrol. Elsevier B.V.; 2015;527: 119–132.

[6] Reggiani P, Rientjes THM. A reflection on the long-term water balance of the Upper Indus Basin. Hydrol Res. 2014; 1–17. 10.2166/nh.2014.060

[7] Jain SK, Agarwal PK, Singh VP. The Indus Basin Hydrology and Water Resources of India. 2007.

[8] Mirza UK, Ahmad N, Majeed T, Harijan K. Hydropower use in Pakistan: Past, present and future. Renew Sustain Energy Rev. 2008;12: 1641–1651. 10.1016/j.rser.2007.01.028

[9] Wanders N, Wada Y. Human and climate impacts on the 21st century hydrological drought. J Hydrol. Elsevier B.V.; 2014; 10.1016/j.jhydrol.2014.10.047

[10] Cheema MJM, Immerzeel WW, Bastiaanssen WGM. Spatial quantification of groundwater abstraction in the irrigated indus basin. Groundwater. 2014;52: 25–36. 10.1111/gwat.12027 23441997

[11] Richey AS, Thomas BF, Lo M-H, Reager JT, Famiglietti JS, Voss K, et al Quantifying renewable groundwater stress with GRACE. Water Resour Res. 2015;51: 5219–5238. 10.1002/2015WR017349 26900185 PMC4744761

[12] Gleeson T, Wada Y, Bierkens MFP, van Beek LPH. Water balance of global aquifers revealed by groundwater footprint. Nature. 2012;488: 197–200. 10.1038/nature11295 22874965

[13] De Souza K, Kituyi E, Harvey B, Leone M, Murali KS, Ford JD. Vulnerability to climate change in three hot spots in Africa and Asia: key issues for policy-relevant adaptation and resilience-building research. Reg Environ Chang. 2015;15: 747–753. 10.1007/s10113-015-0755-8

[14] UN. World Population Prospects, 2015 Revision. New York; 2015.

[15] Lutz AF, ter Maat HW, Biemans H, Shrestha AB, Wester P, Immerzeel WW. Selecting representative climate models for climate change impact studies: an advanced envelope-based selection approach. Int J Climatol. 2016;in press.

[16] IPCC. Managing the risks of extreme events and disasters to advance climate change adaptation. Special Report of the Intergovernmental Panel on Climate Change. Field CB, Barros V, Stocker TF, Q. D, Dokken DJ, Plattner G-K, et al., editors. IPCC. 2012. 10.1596/978-0-8213-8845-7

[17] Houze RA, Rasmussen KL, Medina S, Brodzik SR, Romatschke U. Anomalous atmospheric events leading to the summer 2010 floods in Pakistan. Bull Am Meteorol Soc. 2011;92: 291–298. 10.1175/2010BAMS3173.1

[18] Farr TG, Rosen PA, Caro E, Crippen R, Duren R, Hensley S, et al The Shuttle Radar Topography Mission. Rev Geophys. 2007;45.

[19] Archer DR, Fowler HJ. Spatial and temporal variations in precipitation in the Upper Indus Basin, global teleconnections and hydrological implications. Hydrol Earth Syst Sci. 2004;8: 47–61. 10.5194/hess-8-47-2004

[20] Fowler HJ, Archer DR. Conflicting Signals of Climatic Change in the Upper Indus Basin. J Clim. 2006;19: 4276–4293. 10.1175/JCLI3860.1

[21] Bocchiola D, Diolaiuti G. Recent (1980–2009) evidence of climate change in the upper Karakoram, Pakistan. Theor Appl Climatol. 2013;113: 611–641. 10.1007/s00704-012-0803-y

[22] Quincey DJ, Copland L, Mayer C, Bishop M, Luckman A., Belò M. Ice velocity and climate variations for Baltoro Glacier, Pakistan. J Glaciol. 2009;55: 1061–1071. 10.3189/002214309790794913

[23] Palazzi E, Von Hardenberg J, Provenzale A. Precipitation in the Hindu-Kush Karakoram Himalaya: Observations and future scenarios. J Geophys Res Atmos. 2013;118: 85–100. 10.1029/2012JD018697

[24] Cannon F, Carvalho LMV, Jones C, Norris J. Winter westerly disturbance dynamics and precipitation in the western Himalaya and Karakoram: a wave-tracking approach. Theor Appl Climatol. 2015; 10.1007/s00704-015-1489-8

[25] Cannon F, Carvalho LM V., Jones C, Bookhagen B. Multi-annual variations in winter westerly disturbance activity affecting the Himalaya. Clim Dyn. 2014;44: 441–455. 10.1007/s00382-014-2248-8

[26] Hewitt K. Tributary glacier surges: an exceptional concentration at Panmah Glacier, Karakoram Himalaya. J Glaciol. 2007;53: 181–188. 10.3189/172756507782202829

[27] Kääb A, Berthier E, Nuth C, Gardelle J, Arnaud Y. Contrasting patterns of early twenty-first-century glacier mass change in the Himalayas. Nature. 2012;488: 495–8. 10.1038/nature11324 22914167

[28] Gardelle J, Berthier E, Arnaud Y, Kääb A. Region-wide glacier mass balances over the Pamir-Karakoram-Himalaya during 1999–2011. Cryosph. 2013;7: 1263–1286. 10.5194/tc-7-1263-2013

[29] Gardner AS, Moholdt G, Cogley JG, Wouters B, Arendt AA, Wahr J, et al A reconciled estimate of glacier contributions to sea level rise: 2003 to 2009. Science. 2013;340: 852–85277. 10.1126/science.1234532 23687045

[30] Kääb A, Treichler D, Nuth C, Berthier E. Brief Communication: Contending estimates of 2003–2008 glacier mass balance over the Pamir–Karakoram–Himalaya. Cryosph. 2015;9: 557–564. 10.5194/tc-9-557-2015

[31] Quincey DJ, Glasser NF, Cook SJ, Luckman A. Heterogeneity in Karakoram glacier surges. J Geophys Res Earth Surf. 2015;accepted a. 10.1002/2015JF003515

[32] Tahir AA, Chevallier P, Arnaud Y, Ahmad B. Snow cover dynamics and hydrological regime of the Hunza River basin, Karakoram Range, Northern Pakistan. Hydrol Earth Syst Sci. 2011;15: 2275–2290. 10.5194/hess-15-2275-2011

[33] Tahir AA, Chevallier P, Arnaud Y, Ashraf M, Bhatti MT. Snow cover trend and hydrological characteristics of the Astore River basin (Western Himalayas) and its comparison to the Hunza basin (Karakoram region). Sci Total Environ. Elsevier B.V.; 2015;505: 748–761. 10.1016/j.scitotenv.2014.10.065 25461078

[34] Hasson S, Lucarini V, Khan MR, Petitta M, Bolch T, Gioli G. Early 21st century climatology of snow cover for the western river basins of the Indus River System. Hydrol Earth Syst Sci. 2014;18: 4077–4100. 10.5194/hess-18-4077-2014

[35] Mir RA, Jain SK, Saraf AK, Goswami A. Accuracy assessment and trend analysis of MODIS-derived data on snow-covered areas in the Sutlej basin, Western Himalayas. Int J Remote Sens. 2015;36: 3837–3858. 10.1080/01431161.2015.1070320

[36] Kapnick SB, Delworth TL, Ashfaq M, Malyshev S, Milly PCD. Snowfall less sensitive to warming in Karakoram than in Himalayas due to a unique seasonal cycle. Nat Geosci. 2014; 1–7. 10.1038/ngeo2269

[37] Immerzeel WW, Pellicciotti F, Bierkens MFP. Rising river flows throughout the twenty-first century in two Himalayan glacierized watersheds. Nat Geosci. 2013;6: 742–745. 10.1038/ngeo1896

[38] Soncini A, Bocchiola D, Confortola G, Bianchi A, Rosso R, Mayer C, et al Future Hydrological Regimes in the Upper Indus Basin: A Case Study from a High-Altitude Glacierized Catchment. J Hydrometeorol. 2015;16: 306–326. 10.1175/JHM-D-14-0043.1

[39] Sharif M, Archer DR, Fowler HJ, Forsythe N. Trends in timing and magnitude of flow in the Upper Indus Basin. Hydrol Earth Syst Sci. 2013;17: 1503–1516. 10.5194/hess-17-1503-2013

[40] Mukhopadhyay B, Khan A. Boltzmann–Shannon entropy and river flow stability within Upper Indus Basin in a changing climate. Int J River Basin. 2015;13: 87–95.

[41] Mukhopadhyay B, Khan A. Rising river flows and glacial mass balance in central Karakoram. J Hydrol. Elsevier B.V.; 2014;513: 192–203. 10.1016/j.jhydrol.2014.03.042

[42] Palazzi E, von Hardenberg J, Terzago S, Provenzale A. Precipitation in the Karakoram-Himalaya: a CMIP5 view. Clim Dyn. Springer Berlin Heidelberg; 2014; 21–45. 10.1007/s00382-014-2341-z

[43] Turner AG, Annamalai H. Climate change and the South Asian summer monsoon. Nat Clim Chang. Nature Publishing Group; 2012;2: 587–595. 10.1038/nclimate1495

[44] Rajbhandari R, Shrestha AB, Kulkarni A, Patwardhan SK, Bajracharya SR. Projected changes in climate over the Indus river basin using a high resolution regional climate model (PRECIS). Clim Dyn. 2014; 10.1007/s00382-014-2183-8 25346575

[45] Ali S, Li D, Congbin F, Khan F. Twenty first century climatic and hydrological changes over Upper Indus Basin of Himalayan region of Pakistan. Environ Res Lett. IOP Publishing; 2015;10 10.1088/1748-9326/10/1/014007

[46] Mishra V. Climatic uncertainty in Himalayan Water Towers. J Geophys Res Atmos. 2015;120: 2689–2705. 10.1002/2014JD022650

[47] Forsythe N, Fowler HJ, Blenkinsop S, Burton A, Kilsby CG, Archer DR, et al Application of a stochastic weather generator to assess climate change impacts in a semi-arid climate: The Upper Indus Basin. J Hydrol. Elsevier B.V.; 2014;517: 1019–1034. 10.1016/j.jhydrol.2014.06.031

[48] Mathison C, Wiltshire a. J, Falloon P, Challinor A. J. South Asia river flow projections and their implications for water resources. Hydrol Earth Syst Sci. 2015;12: 5789–5840. 10.5194/hessd-12-5789-2015

[49] Bocchiola D, Diolaiuti G, Soncini A, Mihalcea C, D’Agata C, Mayer C, et al Prediction of future hydrological regimes in poorly gauged high altitude basins: the case study of the upper Indus, Pakistan. Hydrol Earth Syst Sci. 2011;15: 2059–2075. 10.5194/hess-15-2059-2011

[50] Nepal S, Shrestha AB. Impact of climate change on the hydrological regime of the Indus, Ganges and Brahmaputra river basins: a review of the literature. Int J Water Resour Dev. 2015; 1–18. 10.1080/07900627.2015.1030494

[51] Terink W, Lutz AF, Simons GWH, Immerzeel WW, Droogers P. SPHY v2.0: Spatial Processes in HYdrology. Geosci Model Dev. 2015;8: 2009–2034. 10.5194/gmd-8-2009-2015

[52] Immerzeel WW, Wanders N, Lutz AF, Shea JM, Bierkens MFP. Reconciling high altitude precipitation with glacier mass balances and runoff. Hydrol Earth Syst Sci. 2015;12: 4755–4784. 10.5194/hessd-12-4755-2015

[53] Bajracharya SR, Shrestha B. The status of glaciers in the Hindu Kush-Himalayan region. ICIMOD; 2011.

[54] Maussion F, Scherer D, Mölg T, Collier E, Curio J, Finkelnburg R. Precipitation Seasonality and Variability over the Tibetan Plateau as Resolved by the High Asia Reanalysis. J Clim. 2014;27: 1910–1927. 10.1175/JCLI-D-13-00282.1

[55] Scherler D, Bookhagen B, Strecker MR. Spatially variable response of Himalayan glaciers to climate change affected by debris cover. Nat Geosci. 2011;4: 156–159. 10.1038/ngeo1068

[56] Hewitt K. Glacier Change, Concentration, and Elevation Effects in the Karakoram Himalaya, Upper Indus Basin. Mt Res Dev. 2011;31: 188–200. 10.1659/MRD-JOURNAL-D-11-00020.1

[57] Mölg T, Maussion F, Scherer D. Mid-latitude westerlies as a driver of glacier variability in monsoonal High Asia. Nat Clim Chang. 2013;4: 68–73. 10.1038/nclimate2055

[58] Barros AP, Kim G, Williams E, Nesbitt SW. Probing orographic controls in the Himalayas during the monsoon using satellite imagery. Nat Hazards Earth Syst Sci. 2004;4: 29–51. 10.5194/nhess-4-29-2004

[59] Hock R. Temperature index melt modelling in mountain areas. J Hydrol. 2003;282: 104–115. 10.1016/S0022-1694(03)00257-9

[60] Paul F, Huggel C, Kääb A. Combining satellite multispectral image data and a digital elevation model for mapping debris-covered glaciers. Remote Sens Environ. 2004;89: 510–518. 10.1016/j.rse.2003.11.007

[61] Bernhardt M, Schulz K. SnowSlide: A simple routine for calculating gravitational snow transport. Geophys Res Lett. 2010;37: L11502 10.1029/2010GL043086

[62] Cheema MJM, Bastiaanssen WGM. Local calibration of remotely sensed rainfall from the TRMM satellite for different periods and spatial scales in the Indus Basin. Int J Remote Sens. 2012;33: 2603–2627. 10.1080/01431161.2011.617397

[63] Immerzeel WW, Pellicciotti F, Shrestha AB. Glaciers as a Proxy to Quantify the Spatial Distribution of Precipitation in the Hunza Basin. Mt Res Dev. 2012;32: 30–38. 10.1659/MRD-JOURNAL-D-11-00097.1

[64] Dahri ZH, Ludwig F, Moors E, Ahmad B, Khan A, Kabat P. An appraisal of precipitation distribution in the high-altitude catchments of the Indus basin. Sci Total Environ. The Authors; 2016;548–549: 289–306. 10.1016/j.scitotenv.2016.01.001 26802357

[65] Yatagai A, Kamiguchi K, Arakawa O, Hamada A, Yasutomi N, Kitoh A. APHRODITE: Constructing a Long-Term Daily Gridded Precipitation Dataset for Asia Based on a Dense Network of Rain Gauges. Bull Am Meteorol Soc. 2012;93: 1401–1415. 10.1175/BAMS-D-11-00122.1

[66] Kääb A, Berthier E, Nuth C, Gardelle J, Arnaud Y, Kaab A, et al Contrasting patterns of early twenty-first-century glacier mass change in the Himalayas. Nature. Nature Publishing Group; 2012;488: 495–498. 10.1038/nature11324 22914167

[67] Lehner B, Verdin K, Jarvis A. New Global Hydrography Derived From Spaceborne Elevation Data. Eos, Trans Am Geophys Union. 2008;89: 93–104. 10.1029/2008EO100001

[68] Pfeffer W, Arendt A a., Bliss A, Bolch T, Cogley JG, Gardner AS, et al The Randolph Glacier Inventory: a globally complete inventory of glaciers. J Glaciol. 2014;60: 537–552. 10.3189/2014JoG13J176

[69] Defourny P, Vancutsem C, Bicheron P, Brockmann C, Nino F, Schouten L, et al. GLOBCOVER: A 300 m global land cover product for 2005 using ENVISAT MERIS time series. Proceedings of ISPRS Commission VII Mid-Term Symposium: Remote Sensing: from Pixels to Processes, Enschede (NL) 8–11 May 2006. 2007. pp. 8–11.

[70] FAO/IIASA/ISRIC/ISSCAS/JRC. Harmonized World Soil Database (version 1.2). Rome, Italy and Laxenburg, Austria; 2012.

[71] Keshavarzi A, Sarmadian F, Sadeghnejad M, Pezeshki P. Developing Pedotransfer Functions for Estimating some Soil Properties using Artificial Neural Network and Multivariate Regression Approaches. ProEnvironment. 2010;3: 322–330.

[72] Hall DK, Riggs GA, Digirolamo NE, Bayr KJ. MODIS Snow-Cover Products. Remote Sens Environ. 2002;83: 88–89.

[73] Bolch T, Pieczonka T, Mukherjee K, Shea J. Brief Communication: Glaciers in the Hunza Catchment (Karakoram) are in balance since the 1970s. Cryosph Discuss. 2016; 1–11. 10.5194/tc-2016-197

[74] Lutz AF, Immerzeel WW, Gobiet A, Pellicciotti F, Bierkens MFP. Comparison of climate change signals in CMIP3 and CMIP5 multi-model ensembles and implications for Central Asian glaciers. Hydrol Earth Syst Sci. 2013;17: 3661–3677. 10.5194/hess-17-3661-2013

[75] Beven K. A manifesto for the equifinality thesis. J Hydrol. 2006;320: 18–36. 10.1016/j.jhydrol.2005.07.007

[76] Pellicciotti F, Buergi C, Immerzeel WW, Konz M, Shrestha AB. Challenges and Uncertainties in Hydrological Modeling of Remote Hindu Kush–Karakoram–Himalayan (HKH) Basins: Suggestions for Calibration Strategies. Mt Res Dev. 2012;32: 39–50. 10.1659/MRD-JOURNAL-D-11-00092.1

[77] Tachikawa T, Hato M, Kaku M, Iwasaki A. Characteristics of ASTER GDEM version 2. Geoscience and Remote Sensing Symposium (IGARSS), IEEE International. 2011. pp. 3657–3660.

[78] Berthier E, Arnaud Y, Kumar R, Ahmad S, Wagnon P, Chevallier P. Remote sensing estimates of glacier mass balances in the Himachal Pradesh (Western Himalaya, India). Remote Sensing of Environment. 2007 pp. 327–338. 10.1016/j.rse.2006.11.017

[79] Huss M. Density assumptions for converting geodetic glacier volume change to mass change. Cryosph. 2013;7: 877–887. 10.5194/tc-7-877-2013

[80] Bookhagen B, Burbank DW. Toward a complete Himalayan hydrological budget: Spatiotemporal distribution of snowmelt and rainfall and their impact on river discharge. J Geophys Res. 2010;115: 1–25. 10.1029/2009JF001426

[81] Immerzeel WW, Droogers P, de Jong SM, Bierkens MFP. Large-scale monitoring of snow cover and runoff simulation in Himalayan river basins using remote sensing. Remote Sens Environ. Elsevier Inc.; 2009;113: 40–49. 10.1016/j.rse.2008.08.010

[82] Doherty J. PEST: Model Independent Parameter Estimation—Fifth Edition of User Manual. Brisbane; 2005.

[83] Taylor KE, Stouffer RJ, Meehl GA. An Overview of CMIP5 and the Experiment Design. Bull Am Meteorol Soc. 2012;93: 485–498. 10.1175/BAMS-D-11-00094.1

[84] Rosenberg J, Davis SJ, Narloch U, Hallegatte S. Climate constraints on the carbon intensity of economic growth. Environ Res Lett. IOP Publishing; 2015;10: 95006 10.1088/1748-9326/10/9/095006

[85] Arora VK, Scinocca JF, Boer GJ, Christian JR, Denman KL, Flato GM, et al Carbon emission limits required to satisfy future representative concentration pathways of greenhouse gases. 2011;38: 3–8. 10.1029/2010GL046270

[86] Sperber KR, Annamalai H, Kang IS, Kitoh A, Moise A, Turner A, et al The Asian summer monsoon: An intercomparison of CMIP5 vs. CMIP3 simulations of the late 20th century. Clim Dyn. 2013;41: 2711–2744. 10.1007/s00382-012-1607-6

[87] Sperber KR, Annamalai H. The use of fractional accumulated precipitation for the evaluation of the annual cycle of monsoons. Clim Dyn. 2014;43: 3219–3244. 10.1007/s00382-014-2099-3

[88] Sorg A, Huss M, Rohrer M, Stoffel M. The days of plenty might soon be over in glacierized Central Asian catchments. Environ Res Lett. IOP Publishing; 2014;9: 8 10.1088/1748-9326/9/10/104018

[89] van Pelt SC, Beersma JJ, Buishand TA, van den Hurk BJJM, Kabat P. Future changes in extreme precipitation in the Rhine basin based on global and regional climate model simulations. Hydrol Earth Syst Sci. 2012;16: 4517–4530. 10.5194/hess-16-4517-2012

[90] Arnell NW. Climate change and global water resources. Glob Environ Chang. 1999;9: S31–S49.

[91] Kay AL, Davies HN, Bell VA, Jones RG. Comparison of uncertainty sources for climate change impacts: flood frequency in England. Clim Change. 2008;92: 41–63. 10.1007/s10584-008-9471-4

[92] Kraaijenbrink P. Advanced Delta Change method. KNMI De Bilt, The Netherlands; 2013.

[93] Hempel S, Frieler K, Warszawski L, Schewe J, Piontek F. A trend-preserving bias correction—the ISI-MIP approach. Earth Syst Dyn. 2013;4: 219–236. 10.5194/esd-4-219-2013

[94] Maurer EP, Pierce DW. Bias correction can modify climate model simulated precipitation changes without adverse effect on the ensemble mean. Hydrol Earth Syst Sci. 2014;18: 915–925. 10.5194/hess-18-915-2014

[95] Gobiet A, Suklitsch M, Heinrich G. The effect of empirical-statistical correction of intensity-dependent model errors on the temperature climate change signal. Hydrol Earth Syst Sci. 2015;19: 4055–4066.

[96] Ehret U, Zehe E, Wulfmeyer V, Warrach-Sagi K, Liebert J. Should we apply bias correction to global and regional climate model data? (HESS Opinions). Hydrol Earth Syst Sci. 2012;16: 3391–3404. 10.5194/hess-16-3391-2012

[97] Gumbel EJ. The return period of flood flows. Ann Math Stat. 1941;12: 163–190.

[98] Zhang Y, Liu S, Ding Y. Observed degree-day factors and their spatial variation on glaciers in western China. Ann Glaciol. 2006;43: 301–306. 10.3189/172756406781811952

[99] Wagnon P, Vincent C, Arnaud Y, Berthier E, Vuillermoz E, Gruber S, et al Seasonal and annual mass balances of Mera and Pokalde glaciers (Nepal Himalaya) since 2007. Cryosph. 2013;7: 1769–1786. 10.5194/tc-7-1769-2013

[100] MacDonald MK, Pomeroy JW, Pietroniro a. On the importance of sublimation to an alpine snow mass balance in the Canadian Rocky Mountains. Hydrol Earth Syst Sci. 2010;14: 1401–1415. 10.5194/hess-14-1401-2010

[101] Strasser U, Bernhardt M, Weber M, Liston GE, Mauser W. Is snow sublimation important in the alpine water balance? Cryosph. 2008;2: 53–66.

[102] Lenaerts JTM, van den Broeke MR, Déry SJ, König-Langlo G, Ettema J, Munneke PK. Modelling snowdrift sublimation on an Antarctic ice shelf. Cryosph. 2010;4: 179–190. 10.5194/tc-4-179-2010

[103] Nash JE, Sutcliffe JV. River flow forecasting through conceptual models part I—A discussion of principles. J Hydrol. 1970;10: 282–290.

[104] Rangwala I, Miller JR. Climate change in mountains: A review of elevation-dependent warming and its possible causes. Clim Change. 2012;114: 527–547. 10.1007/s10584-012-0419-3

[105] Pepin N, Bradley RS, Diaz HF, Baraer M, Caceres EB, Forsythe N, et al Elevation-dependent warming in mountain regions of the world. Nat Clim Chang. 2015;5: 424–430. 10.1038/nclimate2563

[106] Knutti R, Sedláček J. Robustness and uncertainties in the new CMIP5 climate model projections. Nat Clim Chang. 2012;3: 369–373. 10.1038/nclimate1716

[107] Radić V, Bliss A, Beedlow a. C, Hock R, Miles E, Cogley JG. Regional and global projections of twenty-first century glacier mass changes in response to climate scenarios from global climate models. Clim Dyn. 2014;42: 37–58. 10.1007/s00382-013-1719-7

[108] Zhao L, Ding R, Moore JC. Glacier volume and area change by 2050 in high mountain Asia. Glob Planet Change. 2014;122: 197–207. 10.1016/j.gloplacha.2014.08.006

[109] Viste E, Sorteberg A. Snowfall in the Himalayas: an uncertain future from a little-known past. Cryosph. 2015;9: 1147–1167. 10.5194/tc-9-1147-2015

[110] Khan F, Pilz J, Amjad M, Wiberg DA. Climate variability and its impacts on water resources in the Upper Indus Basin under IPCC climate change scenarios. Int J Glob Warm. 2015;8: 46–69. 10.1504/IJGW.2015.071583

[111] Bliss A, Hock R, Radić V. Global response of glacier runoff to twenty-first century climate change. J Geophys Res Earth Surf. 2014;119: 1–14. 10.1002/2013JF002931

[112] Ramesh K V, Goswami P. Assessing reliability of regional climate projections: the case of Indian monsoon. Nat Sci reports. 2014;4: 4071 10.1038/srep04071 24518919 PMC3921638

[113] Biemans H, Speelman LH, Ludwig F, Moors EJ, Wiltshire a J, Kumar P, et al Future water resources for food production in five South Asian river basins and potential for adaptation—A modeling study. Sci Total Environ. 2013;468–469: S117–S131. 10.1016/j.scitotenv.2013.05.092 23928370

[114] Cannon AJ. Selecting GCM Scenarios that Span the Range of Changes in a Multimodel Ensemble : Application to CMIP5 Climate Extremes Indices. J Clim. 2014;28: 1260–1267. 10.1175/JCLI-D-14-00636.1

[115] Wilcke R, Bärring L. Selecting regional climate scenario for impact modelling studies. Environ Model Softw. 2016;78: 191–201. 10.1016/j.envsoft.2016.01.002

[116] Themeßl MJ, Gobiet A, Leuprecht A. Empirical-statistical downscaling and error correction of daily precipitation from regional climate models. Int J Climatol. 2011;31: 1530–1544. 10.1002/joc.2168

[117] Leander R, Buishand TA. Resampling of regional climate model output for the simulation of extreme river flows. J Hydrol. 2007;332: 487–496. 10.1016/j.jhydrol.2006.08.006

[118] Bordoy R, Burlando P. Bias correction of regional climate model simulations in a region of complex orography. J Appl Meteorol Climatol. 2013;52: 82–101. 10.1175/JAMC-D-11-0149.1

[119] Deque M. Frequency of precipitation and temperature extremes over France in an anthropogenic scenario: Model results and statistical correction according to observed values. Glob Planet Change. 2007;57: 16–26. 10.1016/j.gloplacha.2006.11.030

[120] Biemans H, Hutjes RWA, Kabat P, Strengers BJ, Gerten D, Rost S. Effects of Precipitation Uncertainty on Discharge Calculations for Main River Basins. J Hydrometeorol. 2009;10: 1011–1025. 10.1175/2008JHM1067.1

[121] Koppes M, Rupper S, Asay M, Winter-Billington A. Sensitivity of glacier runoff projections to baseline climate data in the Indus River basin. Front Earth Sci. 2015;3: 1–14. 10.3389/feart.2015.00059

[122] Parajka J, Viglione A, Rogger M, Salinas JL, Sivapalan M, Blöschl G. Comparative assessment of predictions in ungauged basins-Part 1: Runoff-hydrograph studies. Hydrol Earth Syst Sci. 2013;17: 1783–1795.

[123] Ragettli S, Pellicciotti F, Bordoy R, Immerzeel WW. Sources of uncertainty in modeling the glacio-hydrological response of a Karakoram watershed to climate change. Water Resour Res. 2013;49: 1–19. 10.1002/wrcr.20450

[124] Nuimura T, Sakai A, Taniguchi K, Nagai H, Lamsal D, Tsutaki S, et al The GAMDAM Glacier Inventory: a quality controlled inventory of Asian glaciers. Cryosph. 2015;9: 849–864. 10.5194/tc-9-849-2015

[125] Bahr DB, Meier MF, Peckham SD. The physical basis of glacier volume-area scaling. J Geophys Res. 1997;102: 20355–20362.

[126] Frey H, Machguth H, Huss M, Huggel C, Bajracharya S, Bolch T, et al Estimating the volume of glaciers in the Himalayan-Karakoram region using different methods. Cryosph. 2014;8: 2313–2333. 10.5194/tc-8-2313-2014

[127] Qureshi AS. Water Management in the Indus Basin in Pakistan: Challenges and Opportunities. Mt Res Dev. 2011;31: 252–260. 10.1659/MRD-JOURNAL-D-11-00019.1

[128] Siddiqi A, Wescoat JL, Humair S, Afridi K. An empirical analysis of the hydropower portfolio in Pakistan. Energy Policy. Elsevier; 2012;50: 228–241. 10.1016/j.enpol.2012.06.063

